# Transcriptional regulation of *Lonicera japonica* Thunb. during flower development as revealed by comprehensive analysis of transcription factors

**DOI:** 10.1186/s12870-019-1803-1

**Published:** 2019-05-14

**Authors:** Tantan Wang, Bingxian Yang, Qijie Guan, Xi Chen, Zhuoheng Zhong, Wei Huang, Wei Zhu, Jingkui Tian

**Affiliations:** 1 Key Laboratory for Biomedical Engineering of Ministry of Education, College of Biomedical Engineering & Instrument Science, Zhejiang University, Hangzhou, 310027 People’s Republic of China; 2 College of Life Science, Zhejiang Sci-Tech University, Hangzhou, 310018 People’s Republic of China; 3 Zhejiang-Malaysia Joint Research Center for Traditional Medicine, Zhejiang University, Hangzhou, 310027 People’s Republic of China

**Keywords:** *L.japonica*, Flower development, Transcription factor, Hormone, Senescence

## Abstract

**Background:**

*Lonicera japonica* Thunb. flower has been used for the treatment of various diseases for a long time and attracted many studies on its potential effects. Transcription factors (TFs) regulate extensive biological processes during plant development. As the restricted reports of *L. japonica* on TFs, our work was carried out to better understand the TFs’ regulatory roles under different developmental stages in *L. japonica*.

**Results:**

In this study, 1316 TFs belonging to 52 families were identified from the transcriptomic data, and corresponding expression profiles during the *L. japonica* flower development were comprehensively analyzed. 917 (69.68%) TFs were differentially expressed. TFs in bHLH, ERF, MYB, bZIP, and NAC families exhibited obviously altered expression during flower growth. Based on the analysis of differentially expressed TFs (DETFs), TFs in MYB, WRKY, NAC and LSD families that involved in phenylpropanoids biosynthesis, senescence processes and antioxidant activity were detected. The expression of *MYB114* exhibited a positive correlation with the contents of luteoloside; Positive correlation was observed among the expression of *MYC12*, *chalcone synthase (CHS)* and *flavonol synthase (FLS)*, while negative correlation was observed between the expression of *MYB44* and the synthases; The expression of *LSD1* was highly correlated with the expression of *SOD* and the total antioxidant capacity, while the expression of *LOL1* and *LOL2* exhibited a negative correlation with them; Many TFs in NAC and WRKY families may be potentially involved in the senescence process regulated by hormones and reactive oxygen species (ROS). The expression of *NAC19*, *NAC29*, and *NAC53* exhibited a positive correlation with the contents of ABA and H_2_O_2_, while the expression of *WRKY53*, *WRKY54*, and *WRKY70* exhibited a negative correlation with the contents of JA, SA and ABA.

**Conclusions:**

Our study provided a comprehensive characterization of the expression profiles of TFs during the developmental stages of *L. japonica*. In addition, we detected the key TFs that may play significant roles in controlling active components biosynthesis, antioxidant activity and flower senescence in *L. japonica*, thereby providing valuable insights into the molecular networks underlying *L. japonica* flower development.

**Electronic supplementary material:**

The online version of this article (10.1186/s12870-019-1803-1) contains supplementary material, which is available to authorized users.

## Background

*Lonicera japonica* Thunb. (Caprifoliaceae), a medicinal plant, has long been used in traditional Chinese medicine for the treatment of various diseases, such as influenza, cold, fever, and infections [1]. Modern pharmacological studies have proved that the extracts of *L. japonica* exhibit therapeutic potency for many biological and pharmacological activities, such as anti-inflammatory [2], antiviral [3], antibacterial, antioxidant [4], hepatoprotective and anti-tumor [5, 6]. Additionally, recent studies indicated that polysaccharides from *L. japonica* flower buds show hypoglycemic and hypolipidemic effects on streptozotocin-induced diabetic rats, and neuroprotective effects on cerebral ischemia-reperfusion injuries in rats [7, 8]. These findings demonstrated that, *L. japonica* has received much interest in recent years in specialized pharmacological research studies.

*L. japonica* is a perennial, evergreen and twining vine which has double-tongued flowers that open white and fade to yellow [9]. The main components of *L. japonica* include essential oils, phenolic acids, flavone, triterpenoid saponins, iridoids, and inorganic elements, which are considered to be closely related to its pharmacological effects [5]. In *L. japonica*, the accumulation of some active components is various during floral development. For example, the contents of chlorogenic acid (CGA) and luteoloside, the standard chemicals for evaluating the chemical quality of *L. japonica* [10], are highest in slightly white alabastrum, while lower in other developmental stages [11]. Although studies have been conducted to analyze the biosynthesis of active compounds by transcriptome [12–14], the regulatory mechanism of secondary metabolic pathways and the physiological processes during different developmental stages of *L. japonica* remain largely unknown.

The expression levels of genes involved in secondary metabolism are often regulated through developmental, environmental or hormonal processes [15]. As sequence-specific DNA-binding proteins, transcription factors (TFs) function by interacting with regulatory regions and modulating the rate of transcriptional initiation in target genes [16]. Publications showed that several families of TFs play important roles in controlling the biosynthesis and accumulation of secondary metabolites [17]. In *Arabidopsis* MYB family, AtMYB12, AtMYB114, and AtMYB90 are involved in regulating anthocyanin biosynthesis via activation of the entire phenylpropanoid pathway [18, 19]. MYB and bHLH TFs combine with WD40 proteins to form MYB–bHLH–WDR protein complexes (MBW), which regulate flavonoid metabolism processes [20]. However, transcriptomic research that focused on TFs in *L. japonica* is still very limited.

Furthermore, TFs regulate physiological and developmental phenotypes by interacting with plant hormones [21]. Flower development is determined by the combined action of multiple pathways, which involve floral homeotic genes and hormone signaling molecules [22]. TFs and phytohormones including auxins, gibberellin (GA), abscisic acid (ABA), salicylic acid (SA) and jasmonic acid (JA), all play important roles in flower development [23]. For examples, the expression of TF NAC19 is strongly induced by ABA and slightly induced by JA, and NAC19 is involved in mediating programmed cell death (PCD) by reactive oxygen species (ROS) accumulation in plant cells [24]; WRKY53 is modulated by the JA and SA equilibrium in a complex TF signaling network that regulates plant senescence [25]. Yet relevant researches for exploring the physiological and developmental regulation during *L.japonica* growth remain scarce.

In this study, sample materials were collected from *L. japonica* flowers at representative five different developmental stages: the young alabastrum stage (S1), the green alabastrum stage (S2), the whole white alabastrum stage (S3), the silvery flower stage (S4), and the golden flower stage (S5) [26]. In order to investigate the regulation roles of TFs on secondary metabolism and development in *L. japonica* flower, the contents of CGA and luteoloside were measured, and TFs were identified and functionally categorized from RNA-seq data with bioinformatics techniques. What’s more, DETFs (differentially expressed TFs) were clustered based on their expression profiles, and functional annotation in each sub-cluster was performed. Correlation analysis and biological experiments were carried out to confirm the results of TFs analysis.

## Materials and methods

### Plant materials

Seeds of *Lonicera japonica* were purchased from Miaopu Seeds Limited Company (Hangzhou, China). More than 100 of *L. japonica* plants were grown in the Pingyi cultivation base (Shandong Province, China) without exposure to extreme drought, plant diseases, and insect pests. After being authenticated by Professor Lin Zhang at Zhejiang Sci-Tech University, China, flowers of *L. japonica* from five different developmental stages (the juvenile bud stage (S1), the third green stage (S2), the complete white stage (S3), the silver flowering stage (S4), and the gold flowering stage (S5)) were collected in late May. During sample collection, flowers from at least 10 plants were mixed and regarded as one biological replicate representing each stage, and three independent replicates were performed. Flower materials were frozen in liquid nitrogen immediately after collection and then stored at − 80 °C.

### Determination of CGA and luteoloside contents by HPLC

*L. japonica* flower samples were ground in liquid nitrogen and lyophilized. Then, an accurately weighed powder sample (0.010 g) was suspended in 1 mL MeOH, ultrasonically extracted for 1 h, centrifuged at 13,000 g for 5 min and transferred the supernatant. Repeat the extraction step and combine the two supernatants. The extracting solution was concentrated and re-solubilized in 1 mL MeOH, which resulted in samples for CGA and luteoloside contents measuring.

To determine the concentration, the following procedures were performed on Waters 2695 Alliance HPLC system (USA), which equipped with a photodiode array detector, an online degasser and an autosampler for solvent delivery. An aliquot of sample (10 μL) was injected into Waters symmetry C18 column (250 mm × 4.6 mm I.D 5 μm) with a flow rate of 1 mL/min at 20 °C. The mobile phase was composed of 0.05% phosphoric acid in water (A) and 100% acetonitrile (B). The gradients were as follows: 0 min, 10% B; 50 min, 20% B; 60 min, 35% B; 70 min, 95% B. Spectra were measured at wavelength of 327 nm and 350 nm. Peak area was used for quantification.

Preparation of the standard calibration curves: Chlorogenic acid and luteoloside were purchased from Yuanye Biotechnology Co. (Shanghai, China). Calibration curves were constructed using the standard solutions diluted in MeOH at five different concentrations: CGA (0.06, 0.12, 0.18, 0.3, and 0.42 mg/mL), luteoloside (1.5, 3, 4.5, 9, and 18 μg/mL). The regression equations for CGA and luteoloside were y = 309,521x - 203,748 (r^2^ = 0.9991) and y = 1697.2x - 19,711 (r^2^ = 0.9989), respectively.

### RNA isolation and library construction

RNA isolation and library construction were performed according to the method described by Yang et al. (2017) [27]. In brief, a portion (100 mg) of each sample was ground to powder in liquid nitrogen. Total RNA was isolated using RNeasy Plant Mini kit (Qiagen, Hilden, Germany). The quantity and quality of RNA were determined using Agilent 2100 Bioanalyzer (Agilent Technologies, Palo Alto, CA, USA). The poly (A) mRNA was isolated from total RNA samples with Magnetic Oligo (dT) Beads (Illumina, San Diego, CA, USA) and used for mRNA-sequencing library construction. cDNA was synthesized using the fragmented mRNA with the incorporation of reverse transcriptase for end-repair, followed by a single ‘A’ base addition. mRNA-Sequencing Sample Preparation Kit (Illumina) was used to prepare the DNA fragments for ligation to the adapters. After purification, the cDNA fragments (200 ± 25 bp) were excised and retrieved. Polymerase chain reaction (PCR) was performed to selectively enrich and amplify the cDNA fragments. Libraries were prepared from a 150–200 bp size-selected fraction following adapter ligation and agarose gel separation.

### Sequencing, de novo assembly and annotation

The mRNA-sequencing libraries were sequenced using the Hiseq 2000 sequencing platform (Illumina). Raw reads were filtered using BWA [28], with a quality threshold of 30. The Trinity program was used to assemble the clean reads and obtain non-redundant unigenes [29]. Annotation of the assembled unigenes was performed by searching against the NCBI non-redundant protein (NR), Swiss-Prot, TrEMBL, and Pfam databases. The search was conducted using BLAST with an E-value cut-off of 1e-5. Functional annotation by gene ontology terms (GO, http://www.geneontology.org) was carried out using the Blast2GO software [30].

### Identification and expression analysis of TFs

The amino sequences of TFs in *Arabidopsis thaliana* and *Coffea canephora* were downloaded from the PlantTFDB database [31]. For TF identification in *L. japonica,* Blast+ was used for sequence alignment with the identity cut-off threshold of 30% and E-value of 1e-5 [32]. Candidates that contain DNA binding domains were recognized by GO annotation for the final TF identification. Differentially expressed TFs (DETFs) between samples were identified using the value of Fragments Per Kilobase of transcript Per Million fragments mapped (FPKM) with |log2(fold change)| > 1, *p* value ≤0.05 and q value ≤0.05 [33]. The Multiexperiment Viewer software (v4.9) was employed to exhibit the expression profiles of DETFs by clustering [34]. DETFs were clustered by K-means method. Using the total TFs in *L. japonica* as reference, GO enrichment analysis of DETFs was performed using Agrigo (http://bioinfo.cau.edu.cn/agriGO/) with hypergeometric test and FDR cut-off of 0.05 [35].

### Measurement of endogenous hormones in *L. japonica*

Samples were prepared using the method of Pan et al. with minor modification [36]. Firstly, 50 mg sample was ground into fine power in liquid nitrogen. Then, 500 μL of 1-propanol/H2O/concentrated HCl (2: 1: 0.002, v: v: v) was added, and the mixture was agitated for 30 min at 4 °C. Next, the solution was centrifuged at 13,000 g for 5 min at 4 °C. 1 mL of CH_2_Cl_2_ was added to the isolated upper layer, and the new mixture was agitated for 30 min at 4 °C then centrifuged at 13,000 g for 5 min at 4 °C. The lower layer (~ 1 mL) was concentrated and re-solubilized in 0.2 mL of 50% MeOH, which resulted in the samples for hormones levels determination.

The following determination was performed on Agilent 6460 Triple Quadrupole LC-MS/MS (Agilent Technologies, Technologies, Palo Alto, CA, USA) with an electrospray ESI (Agilent Technologies). An aliquot (20 μL) was injected into Zobax XDB C18 column (2.1 mm × 150 mm × 3.5 μm, Agilent Technologies) with a flow rate of 0.3 mL/min. Peak abundance was used for ABA, JA, and SA quantification. The mobile phase was 0.1% formic acid in water (A) and 100% MeOH (B). The gradient was as follows: 0.00 min, 60% A (40% B); 1.5 min, 60% A (40% B); 8 min, 0% A (100% B); 8.5 min, 60% A (40% B).

### Measurement of hydrogen peroxide (H_2_O_2_) and total antioxidant capacity in *L. japonica*

To measure the H_2_O_2_ content and the total antioxidant capacity, 100 mg sample was grounded with 0.9 mL deionized distilled H_2_O (dd H_2_O) on ice. The resulting mixture was centrifuged at 10,000 g for 10 min at 4 °C, and the upper layer was isolated for the subsequent process. Protein concentration was determined using the Bradford assay with bovine serum albumin as standard [37]. The Hydrogen Peroxide Assay Kit (Nanjing Jiancheng Bioengineering Institute, Nanjing, China) and the Total Antioxidant Capacity (T-AOC) Assay Kit (Nanjing Jiancheng Bioengineering Institute, Nanjing, China) were used for measuring the H_2_O_2_ content and the total antioxidant capacity.

### Statistical analysis

Significant differences between contents of CGA, luteoloside, hormones and H_2_O_2_, and total antioxidant capacity were calculated using a one-way ANOVA analysis with a Turkey test and a significance level at α = 0.05 in SPSS software. All expression analyses were performed in three replicates. Reported values represent arithmetic averages of three replicates. Data was expressed as mean plus or minus standard deviation (mean ± SD).

## Results

### Contents of CGA and luteoloside in different developmental stages of *L.japonica* flower

**Fig. 1:**
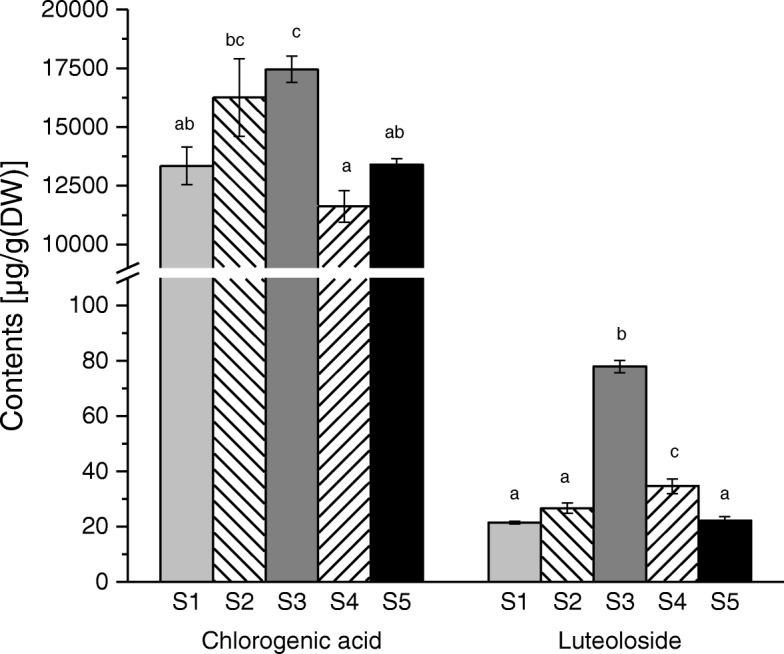
Contents of CGA and luteoloside in five different developmental stages of the *L. japonica* flower. Data is shown as the mean ± SD from three independent experiment replicates. a, b, c indicate significant changes measured by one-way ANOVA analysis (α < 0.05)

The contents of CGA and luteoloside in S1, S2, S3, S4 and S5 of *L.japonica* flower were measured by HPLC (Fig. 1). The morphological photos corresponding to each stage were shown in Additional file 1: Figure S1. According to the results, the content of CGA was higher at S2 and S3, while was lowest at S4. The content of luteoloside at S3 was the highest, and about three times higher than other stages. Overall, the content of CGA exhibited an increase from S1 to S3, while reached its minimum at S4, and then increased again at S5; The content of luteoloside increased from S1 to S3, followed by a decrease from S3 to S5.

### Sequencing, assembly and annotation of *L.japonica* transcriptome

To acquire comprehensive TF expression profiles of *L.japonica* flowers at five stages of development*,* transcriptome sequencing and analysis were performed. Clean pair-end reads were obtained after removal of adaptor sequences and low-quality reads. The Trinity program was used for de novo assembly of all clean reads, which yielded a total of 43,689 unigenes with an average length of 1033 bp, N50 of 1612 bp, and GC content of 41.49% (Table 1). The assembled unigenes were annotated by common databases including the Swiss-Prot, TrEBML, NR, Pfam and GO, to which approximately 39.00, 65.30, 65.80, 53.20, and 54.60% of unigenes were mapped respectively (Table 2). Among the total unigenes, 15,585 (36%) were annotated by all the five databases. The number of unigenes annotated by only one database were 0, 19, 227, 0, 0 for Swiss-Prot, TrEBML, NR, Pfam and GO, individually (Additional file 1: Figure S2).

**Table 1:** Assembly summary of transcriptomic data

Assembly	Number
Total unigenes generated	43,689
N_50_ length (bp)	1612
Average unigenes length (bp)	1033
GC(%)	41.49

**Table 2:** Annotation results of the unigenes

Unigene	Swiss_Prot	TrEBML	NR	Pfam	GO
43,689	17,033	28,543	28,748	23,262	23,867
100%	39.00%	65.30%	65.80%	53.20%	54.60%

### Identification and classification of TFs

**Fig. 2:**
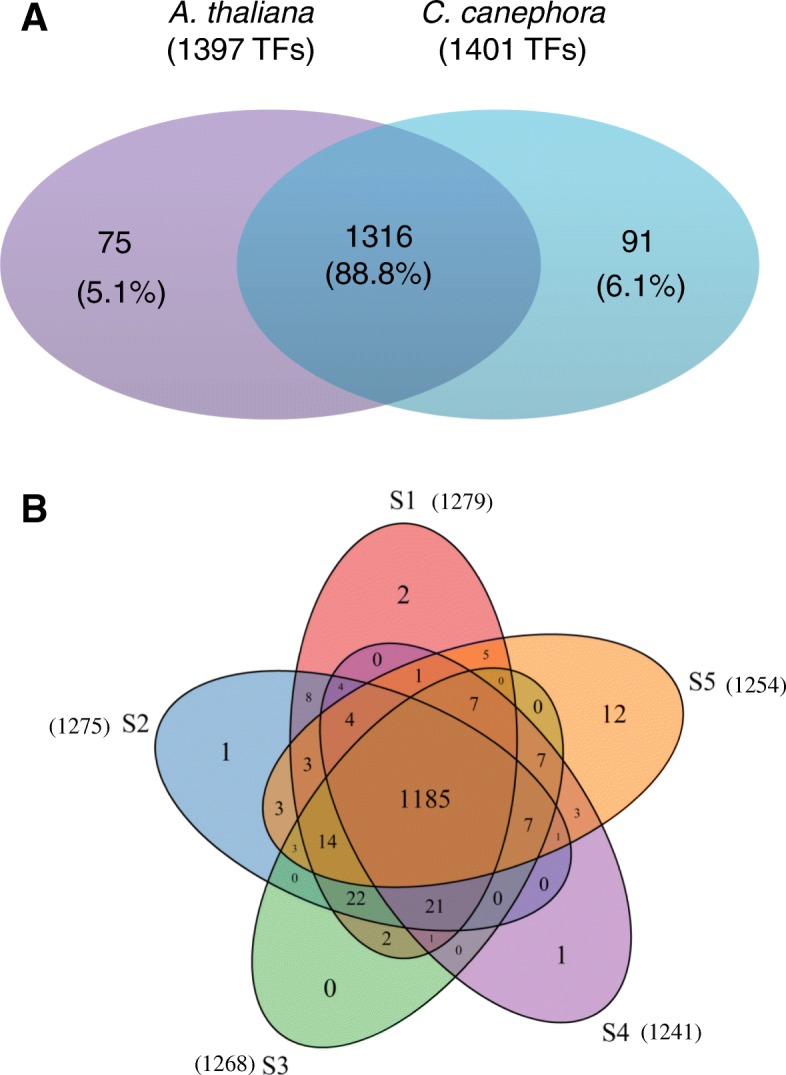
Identification and distribution of TFs. **a** Assembled *L. japonica* unigenes were aligned with the amino sequences of TFs in *A. thaliana* and *C. canephora* by Blast+ with an E-value cut-off of 1e-5. **b** Distribution of TFs in five developmental stages of *L. japonica* flower. The TFs identified were based on the intersection results of *A. thaliana* and *C. canephora* shown in (**a**), a total of 1316 TFs were detected. The five stages were the juvenile bud stage (S1), the third green stage (S2), the complete white stage (S3), the silver flowering stage (S4), and the gold flowering stage (S5)

Many TFs have been found to play important roles in plant growth [38]. To detect the biological functions of TFs involved in the development of *L. japonica* flower*,* TFs expressing in five different developmental stages were identified. To ensure the accuracy of TF identification and classification, we aligned the unigenes of *L. japonica* with the amino acid sequences of TFs in two species including *A. thaliana* and *C.canephora*. *A. thaliana* is a model plant. *C. canephora* is one of the top species that has the most homologous genes with *L.japonica* in NR annotation, indicating that *C.canephora* may share a close polygenetic relationship with *L. japonica* (Additional file 1: Figure S3). Also, according to Flora of China, *C. canephora* and *L.japonica* both belong to Rubiales Family, which strengthened the close relationship between them [39]. The results showed that, 1391 unigenes were mapped to the amino acid sequences of *A. thaliana*’s TFs, and 1407 unigenes were mapped to the amino acid sequences of *C. canephora*’s TFs. As shown in Fig. 2a, we selected the 1316 unigenes of *L. japonica* which were mapped to both *A. thaliana* and *C.canephora* for further analysis.

**Fig. 3:**
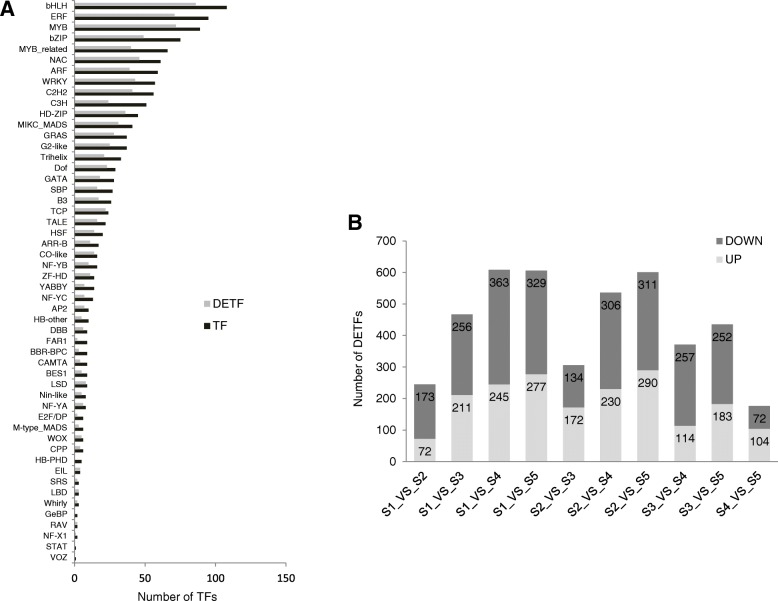
Classification and differential expression of TFs in five stages. **a** Distribution of total TFs and differentially expressed TFs (DETFs) among different TF families. X-axis represents number of TFs. **b** Pairwise comparison of TFs in five developmental stages. Y-axis represents number of DETFs

Among the 1316 TFs, 1279, 1275, 1268, 1241, 1254 TFs were found in S1, S2, S3, S4 and S5, respectively. 1185 TFs were expressed in all five stages, representing 90.04% of the total TFs. S5 contained the most stage-specific TFs (12), which were from TF families including YABBY, GRAS, MIKC_MADS, ERF, HD-ZIP and SBP (Fig. 2b, Table 3). All the analyzed TFs (1316) were classified into 52 families. The five largest families represented were bHLH (108), ERF (95), MYB (89), bZIP (75), MYB_related (66) (Fig. 3a).

**Table 3:** S5 specific TFs

NO.	TF	TF family
1	YAB2	YABBY
2	SCL15	GRAS
3	AGL8	MIKC_MADS
4	ERF3	ERF
5	HAT7	HD-ZIP
6	SPL1	SBP
7	SPL1	SBP
8	ERF3	ERF
9	YAB2	YABBY
10	SPL1	SBP
11	AGLB	MIKC_MADS
12	ERF3	ERF

### Analysis of differentially expressed TFs (DETFs)

To acquire insights into functional and regulatory dynamics during flower development, pairwise differential analysis was conducted on the expression levels of TFs in the five developmental stages. Results showed that 917 TFs, classified into 48 families, were differentially expressed in at least one of the pairwise comparisons among the five flowering stages, representing 69.68% of the total TFs. Most DETFs (86) belonged to the bHLH family, followed by ERF (71), MYB (72), bZIP (49), NAC (46). In addition, expression levels of TFs from some families, such as TCP, CO-like, LSD, EIL were found to fluctuate significantly among the five stages. Lower portions of the TFs in MYB_related, C3H, SBP, FAR1, BBR-BPC and E2F/DP families were differentially expressed. Meanwhile, expression levels of TFs in HB-PHD, GeBP, STAT and VOZ families remained relatively stable throughout development (Fig. 3a).

To further decipher the expression profiles of TFs, the up- or down-regulated TFs in each pairwise comparison were identified (Fig. 3b). The comparison S1_VS_S4, S2_VS_S4, S1_VS_S5, S2_VS_S5 exhibited more DETFs, while S1_VS_S2, S4_VS_S5 showed fewer DETFs. This result indicated that S4 and S5 shared relatively similar situation during *L. japonica* flower development, so were S1 and S2. This observation was consistent with the cluster result shown in Additional file 1: Figure S4. Overall, an increasing number of up-regulated TFs was found along with the growth of *L. japonica*. For example, when expression of TFs in all stages were compared to that in S1, 72 DETFs in S1_VS_S2, 211 DETFs in S1_VS_S3, 245 DETFs in S1_VS_S4, and 277 DETFs in S1_VS_S5. This finding implied TFs might be involved in more activities in flowering development of *L. japonica*.

### Time-course expression of DETFs

**Fig. 4:**
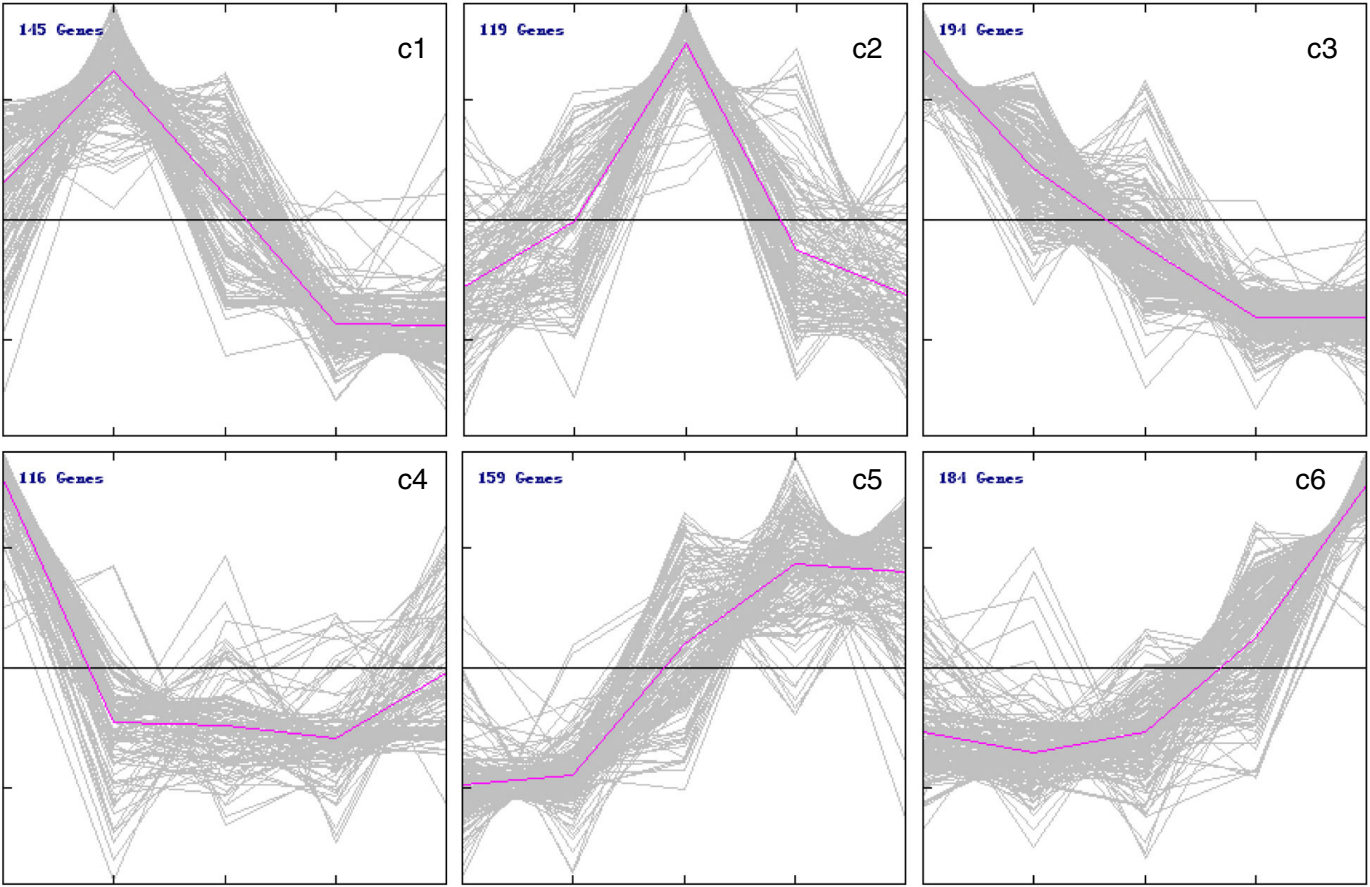
Clusters of differential expression TFs (DETFs). DETFs were clustered and displayed in line chart using Mev. Six clusters were grouped. X-axis represents the five different developmental stages of *L. japonica* flower including S1, S2, S3, S4 and S5. Y-axis represents normalized value of the DETFs’ expression level. The middle black line in each cluster is the zero line and the red line indicates the average expression level. The number in the upper left corner represents the number of TFs in each cluster

To clarify the time-course expression of DETFs in *L. japonica*, the 917 DETFs were grouped into six clusters based on their temporal expression profiles (Fig. 4). Cluster 1 contained 145 TFs that achieved their maximum expression at S2, followed by a decrease with further development. Cluster 2 contained 119 TFs that exhibited an increase in expression level from S1 to S3, followed by a decrease from S3 to S5. Cluster 3 contained 194 TFs that peaked at S1 and then steadily decreased across the five-time points. Cluster 4 was comprised of 116 TFs whose expression decreased sharply from S1 to S2, slowly at S3 and S4, and then increased slightly from S4 to S5. Cluster 5 contained 159 TFs that increased in expression from S1 to S4, and then exhibited a decrease in S5. The expression of TFs belonging to cluster 6 exhibited little changes between S1 and S2, followed by a surge from S3 to S5. Cluster 1 to 6 contained 13.0, 21.2, 15.8, 12.6, 17.3, and 20.1% of all DETFs, respectively. The largest TF family represented in cluster 1, 3 and 5 was bHLH and that in cluster 4 and 6 was NAC. Furthermore, several large TF families such as NAC, ERF, WRKY, MYB, bHLH were found to made up more than half of the TFs in cluster 4. In cluster 6, NAC, ERF, bHLH were the top three most abundant families (Additional file 1: Figure S5).

### GO enrichment analysis of time-course expression patterns

**Fig. 5:**
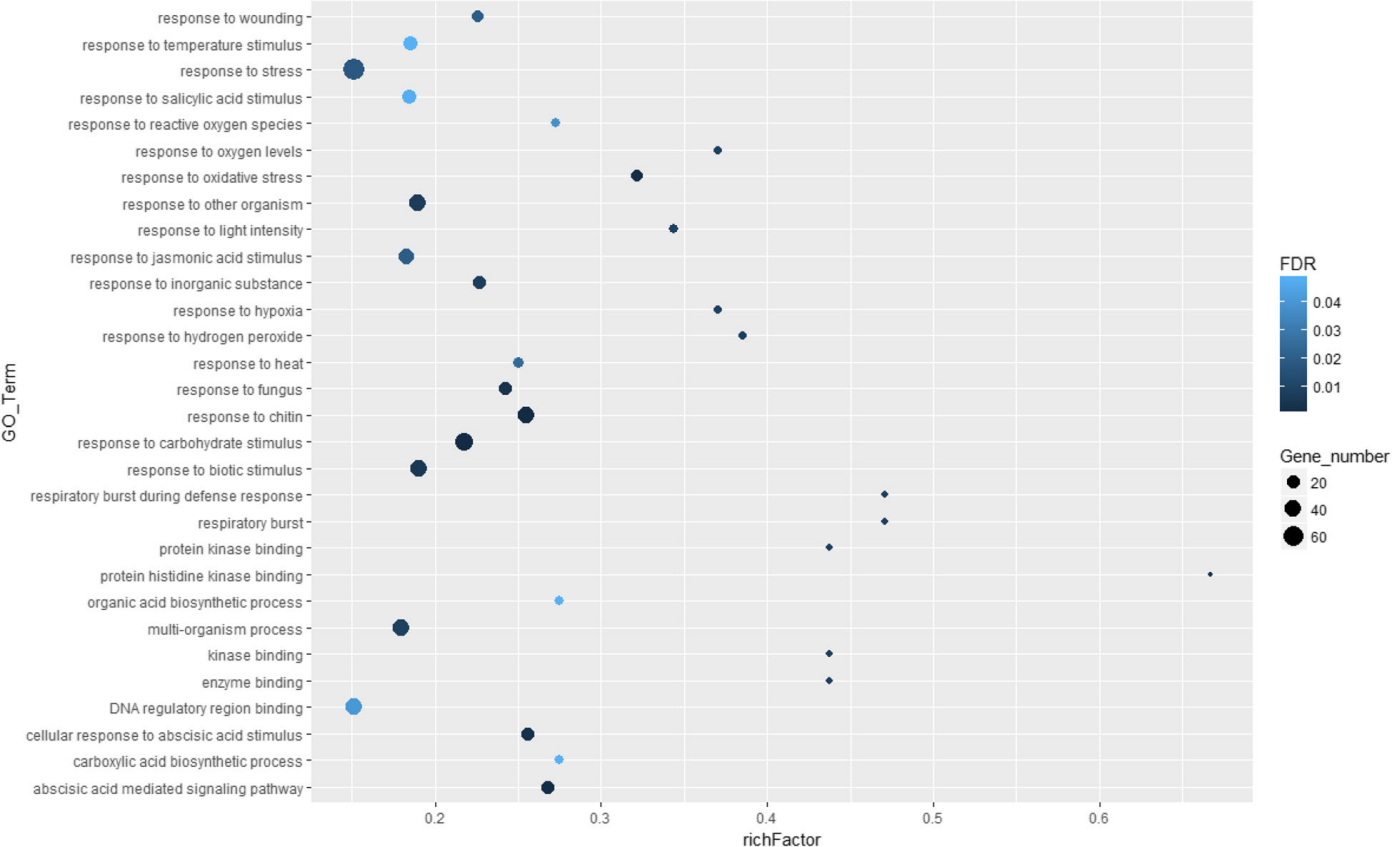
Go enrichment of TFs in cluster 4. GO enrichment analysis was performed using Agrigo with FDR value cut-off of 0.05 and the total TFs in *L. japonica* flower were used as reference. Color of circle in the plot indicates FDR value and size of circle indicates the number of TFs in each GO_Term

**Fig. 6:**
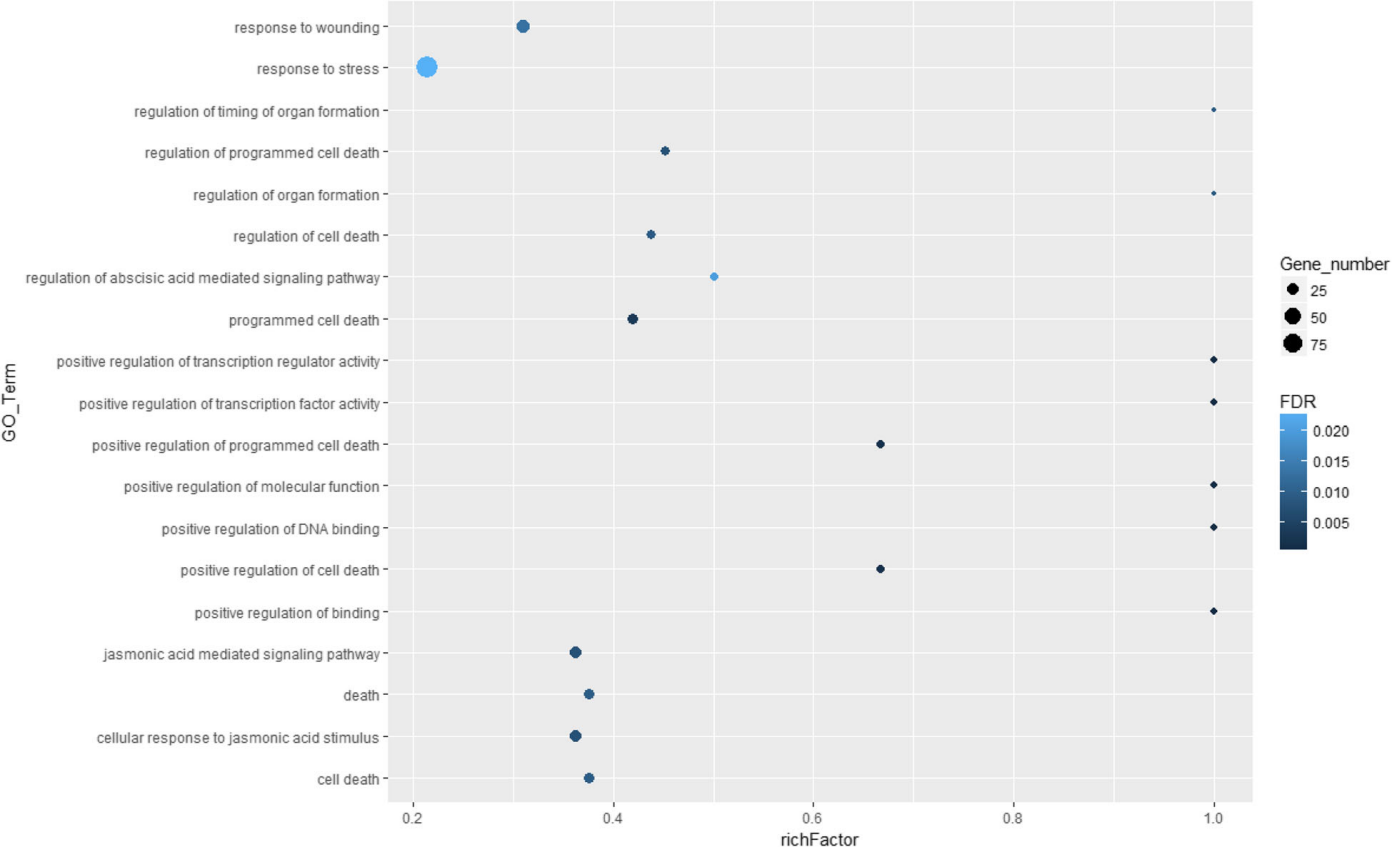
GO enrichment of TFs in cluster 6. GO enrichment analysis was performed using Agrigo with FDR value cut-off of 0.05 and the total TFs in *L. japonica* flower were used as reference. Color of circle in the plot indicates FDR value and size of circle indicates the number of TFs in each GO_Term

To further elucidate the functional mechanism of TFs in mediating flower development, GO enrichment analysis was performed on the six clusters of DETFs. Our results showed that, in cluster 1, there were no significantly enriched GO items; Many TFs in cluster 2 were associated with “protein self-association”; In cluster 3, the significant functions of TFs were “lipid binding” and “cotyledon development”; TFs in cluster 5 might play important roles in “ligase activity” (Additional file 2: Table S1). TFs in cluster 4 and cluster 6 were involved in multiple functions. Specifically, in cluster 4, the results of GO enrichment analysis indicated that TFs in this group were primarily associated with various stimuli, responses and biosynthetic processes, including JA stimulus, SA stimulus, ABA stimulus, carbohydrate stimulus, biotic stimulus, response to ROS, and organic acid biosynthetic process (Fig. 5). In cluster 6, most of the TFs were involved in the regulation of PCD, JA-mediated signaling pathway and ABA-mediated signaling pathway (Fig. 6). These findings demonstrated that the TFs in cluster 4 and cluster 6 might have essential roles in plant hormones, ROS and PCD during the growth and development of the *L. japonica* flower.

### Endogenous hormones measurements

**Fig. 7:**
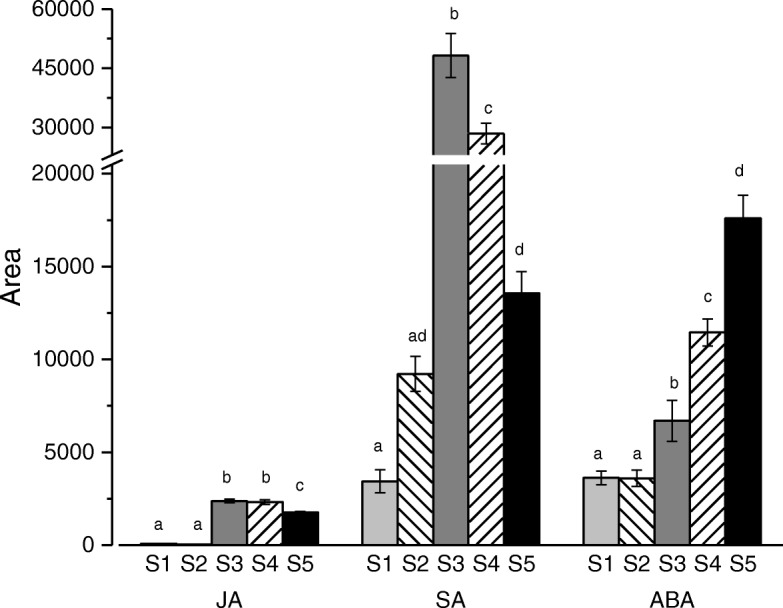
Contents of endogenous hormones including JA, SA, and ABA in different developmental stages of the *L. japonica* flower. Data is shown as mean ± SD from three independent experiment replicates. a, b, c indicate significant changes measured by one-way ANOVA analysis (α < 0.05)

TFs and plant hormones have been shown to influence each other during plant growth in a complex relationship [21]. In order to better understand the interaction between endogenous hormones and TFs in the transcription activities during *L. japonica* flower development, the contents of hormones including JA, SA, and ABA were measured (Fig. 7). The results showed that both JA and SA were found at low concentrations in the early stages of flower development, and then a surge at S3 was observed, followed by a decrease from S4 to S5. The difference between the contents of JA and SA was that, in the last two periods, JA content decreased slightly while that of SA decreased dramatically. The content of ABA reached a plateau at S1 and S2, then steadily increased until a maximum was reached at S5. Moreover, the trend of ABA concentration throughout development was very similar to the expression trend of TFs observed in cluster 6 (Figs. 4 and 7).

### Determination of hydrogen peroxide (H_2_O_2_) content and the total antioxidant capacity

**Fig. 8:**
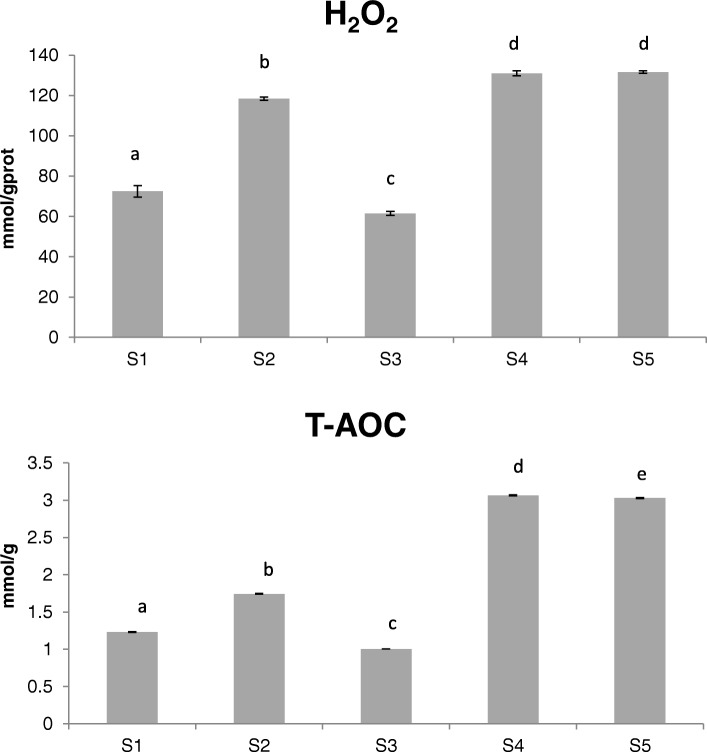
Content of hydrogen peroxide (H_2_O_2_) and the total antioxidant capacity in different developmental stages of the *L. japonica* flower. Data is shown as mean ± SD from three independent experiment replicates. a, b, c, d, e indicate significant changes measured by one-way ANOVA analysis (α < 0.05)

In plants, various abiotic stresses can lead to the formation of ROS, which has been proposed as key inducers of developmental PCD [40]. H_2_O_2_ is one of the main members of ROS, and its effect on PCD has been well studied. Antioxidant system is important in the maintenance of balanced ROS levels [41]. In our research, the TFs in cluster 4 and 6 were found to have a close association with H_2_O_2_ and PCD (Fig. 6 and 7). To investigate the association among them, the content of H_2_O_2_ and the total antioxidant capacity were evaluated (Fig. 8). Our results showed both of them followed a similar trend, with low levels observed at the early stages of flower development, followed by high levels at S4 and S5. In addition, these trends were consistent with the trend of ABA’s content and the expression pattern of TFs in cluster 6 (Figs. 4 and 8).

#### Correlation analysis of specific TFs

**Fig. 9:**
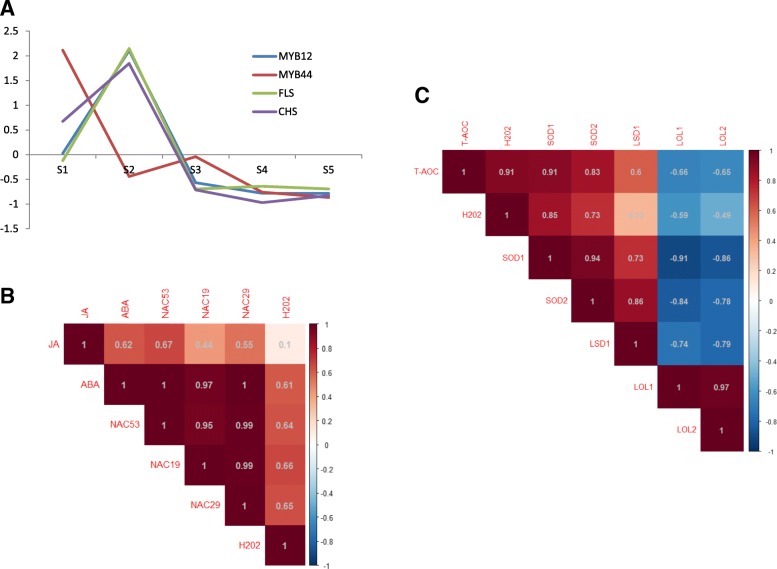
Correlations among TFs, genes, hormones, and H_2_O_2_. The expression of the genes, the contents of hormones and ROS were normalized by “Z-score”. **a** The expression patterns of *MYB12*, *MYB44*, *CHS* and *FLS*, which are involved in flavonoid biosynthesis. **b** The correlations among the expression of *NAC19*, *NAC29* and *NAC53*, the contents of JA, ABA, and H_2_O_2_, which are involved in senescence processes. **c** The correlations among the expression of *LSD1*, *LOL1*, *LOL2* and *SOD*. The number in each square represents the corresponding correlation coefficient. Color intensity is proportional to the correlation coefficient. Positive correlations are displayed in red and negative correlations in blue

Based on the cluster and enrichment results, the key TFs involved in secondary metabolism and *L. japonica* flower development were detected. Figure 9 showed the expression patterns and their correlations. CHS and FLS have been reported as the key enzymes in the flavonol pathway [42]. Our results demonstrated that MYB114, MYB12 and MYB44 might be involved in flavonoid biosynthetic processes (Additional file 3: Table S2). In this study, the expressions of *CHS* and *FLS* exhibited similar time-course patterns to the expression of *MYB12* from S1 to S5, while a reverse trend was observed when compared with the expression of *MYB44* at S1, S2 and S3 (Fig. 9a). Additionally, NAC19, NAC29 and NAC53 might be involved in JA- and ABA-mediated signaling pathways and PCD (Table 4). The expressions of the three TFs in cluster 6 and the contents of ABA and H_2_O_2_ were very consistent, and JA content was also associated with the expression of these TFs (Fig. 9b). Moreover, the expression of *LSD1* and *SOD* was highly correlated, while the expression of *LOL1* and *LOL2* displayed a negative correlation with them (Fig. 9c). In addition, the TF WRKY70 might be involved in ABA mediated signaling pathway, JA stimulus, and SA stimulus. WRKY53 might be involved in JA stimulus, and WRKY54 might be involved in JA stimulus, SA stimulus, and ROS (Table 4). These three TFs belonged to cluster 4 and showed similar expression pattern that exhibited high expression at S1 and followed by lower expression in later stages (Table 4).

**Table 4:** Enrichment results of TFs associated with hormones and PCD in cluster 4 and cluster 6

No.	Family	TF	Access	Species	E-value	Identify	FPKM					Function
							S1	S2	S3	S4	S5	
c4												
1	ERF	ERF028	Q9FJ93	Ath	2.2E-38	71.96	174.02	31.22	26.42	7.45	11.39	ABA-m
2	bHLH	MYC2	Q39204	Ath	3.5E-47	72.66	116.49	27.68	18.57	5.73	10.43	ABA-m
3	MYB	MYB44	Q9FDW1	Ath	5.4E-33	77.78	30.44	5.77	9.63	2.70	1.65	ABA-m
4	C2H2	AZF2	Q9SSW2	Ath	1.4E-10	93.1	124.21	47.16	37.00	21.77	64.54	ABA-m
5	bHLH	BHLH28	Q9LUK7	Ath	8.9E-27	81.43	15.93	2.22	5.37	0.03	0.36	ABA-m
6	WRKY	–	–	–	–	–	331.73	40.78	75.34	6.91	98.37	ABA-m
7	C2H2	AZF2	Q9SSW2	Ath	2.3E-08	82.76	157.64	43.24	26.69	16.78	40.29	ABA-m
8	MYB	MYB44	Q9FDW1	Ath	1.2E-28	74.03	10.08	2.37	0.36	0.00	0.00	ABA-m
9	MYB	MYB44	Q9FDW1	Ath	2.4E-16	78	59.04	10.26	21.91	5.89	4.06	ABA-m
10	MYB	MYB44	Q9FDW1	Ath	1.1E-52	89.52	97.00	36.58	67.57	44.99	66.88	ABA-m
11	ERF	ERF072	Q9M0L0	Ath	5.2E-44	75.42	155.37	9.12	11.01	0.22	0.64	ABA-m
12	MYB	MYB44	Q9FDW1	Ath	2.4E-48	81.9	75.94	16.12	7.40	1.37	2.61	ABA-m
13	WRKY	WRKY40	Q9SAH7	Ath	4E-29	92.19	92.23	19.30	36.11	27.31	49.44	ABA-m
14	WRKY	WRKY70	Q9LY00	Ath	1.6E-17	62.71	51.75	18.71	35.62	40.34	33.77	ABA-m
15	MYB	MYB44	Q9FDW1	Ath	1.1E-50	85.71	146.82	26.67	17.47	5.88	7.90	ABA-m
16	NAC	NAC102	Q8H115	Ath	5.9E-26	90	63.31	18.15	8.28	23.77	51.21	ABA-m
17	NAC	NAC102	Q8H115	Ath	1.6E-51	90.1	30.93	9.31	3.19	8.77	28.20	ABA-m
18	NAC	NAC002	Q39013	Ath	1.7E-21	88.46	200.17	30.53	26.42	32.49	83.49	ABA-m
19	NAC	NAC002	Q39013	Ath	2.3E-44	90.7	601.19	127.24	89.40	102.27	186.80	ABA-m
20	NAC	NAC102	Q8H115	Ath	4.8E-51	90.91	104.53	27.28	22.25	28.75	76.53	ABA-m
21	bZIP	ABF2	Q9M7Q4	Ath	8.5E-38	79	8.86	2.81	1.49	2.44	3.01	ABA-m
22	ERF	ERF042	Q52QU1	Ath	1.5E-09	84.38	154.22	26.46	18.96	1.81	5.19	ABA-m
23	bHLH	BHLH13	Q9LNJ5	Ath	1.1E-86	70.62	24.28	5.51	15.05	12.89	14.44	ABA-m
24	ERF	ERF109	Q9SZ06	Ath	6.7E-25	80.88	64.11	7.22	1.88	0.32	0.81	JA-sti
25	MYB_related	RVE1	F4KGY6	Ath	1.4E-18	86	8.80	2.50	2.97	3.56	4.58	JA-sti
26	ERF	ERF12	Q94ID6	Ath	6.9E-29	92.06	7.44	1.08	2.68	2.84	5.08	JA-sti
2	bHLH	MYC2	Q39204	Ath	3.5E-47	72.66	116.49	27.68	18.57	5.73	10.43	JA-sti
3	MYB	MYB44	Q9FDW1	Ath	5.4E-33	77.78	30.44	5.77	9.63	2.70	1.65	JA-sti
5	bHLH	BHLH28	Q9LUK7	Ath	8.9E-27	81.43	15.93	2.22	5.37	0.03	0.36	JA-sti
10	MYB	MYB44	Q9FDW1	Ath	1.1E-52	89.52	97.00	36.58	67.57	44.99	66.88	JA-sti
8	MYB	MYB44	Q9FDW1	Ath	1.2E-28	74.03	10.08	2.37	0.36	0.00	0.00	JA-sti
9	MYB	MYB44	Q9FDW1	Ath	2.4E-16	78	59.04	10.26	21.91	5.89	4.06	JA-sti
27	ERF	ERF4	Q9LW49	Nsy	2.7E-28	88.06	70.47	37.92	28.64	19.73	39.27	JA-sti
28	MYB	ODO1	Q50EX6	Phy	4.7E-66	86.05	22.68	7.22	6.52	14.36	21.03	JA-sti
12	MYB	MYB44	Q9FDW1	Ath	2.4E-48	81.9	75.94	16.12	7.40	1.37	2.61	JA-sti
29	MYB	ODO1	Q50EX6	Phy	2.2E-72	95.35	10.64	1.88	0.75	0.23	2.01	JA-sti
13	WRKY	WRKY40	Q9SAH7	Ath	4E-29	92.19	92.23	19.30	36.11	27.31	49.44	JA-sti
14	WRKY	WRKY70	Q9LY00	Ath	1.6E-17	62.71	51.75	18.71	35.62	40.34	33.77	JA-sti
30	C3H	AtC3H2	Q9ZWA1	Ath	6.7E-59	82.76	21.60	2.65	3.25	0.14	0.15	JA-sti
15	MYB	MYB44	Q9FDW1	Ath	1.1E-50	85.71	146.82	26.67	17.47	5.88	7.90	JA-sti
16	NAC	NAC102	Q8H115	Ath	5.9E-26	90	63.31	18.15	8.28	23.77	51.21	JA-sti
17	NAC	NAC102	Q8H115	Ath	1.6E-51	90.1	30.93	9.31	3.19	8.77	28.20	JA-sti
18	NAC	NAC002	Q39013	Ath	1.7E-21	88.46	200.17	30.53	26.42	32.49	83.49	JA-sti
31	NAC	NAC102	Q8H115	Ath	1.3E-21	90.38	318.41	81.88	49.04	78.02	101.43	JA-sti
19	NAC	NAC002	Q39013	Ath	2.3E-44	90.7	601.19	127.24	89.40	102.27	186.80	JA-sti
20	NAC	NAC102	Q8H115	Ath	4.8E-51	90.91	104.53	27.28	22.25	28.75	76.53	JA-sti
32	ERF	EREBP1	Q6K7E6	Osa	2.3E-29	86.96	109.31	19.46	24.40	63.24	80.13	JA-sti
33	ERF	ERF4	O80340	Ath	2.7E-30	88.73	15.03	5.03	2.49	1.04	3.03	JA-sti
34	ERF	ERF72	P42736	Ath	4E-27	88.89	41.03	22.00	35.14	13.74	33.80	JA-sti
6	WRKY	–	–	–	–	–	331.73	40.78	75.34	6.91	98.37	JA-sti
35	WRKY	WRKY54	Q93WU8	Ath	2.1E-13	77.27	144.39	27.50	27.56	10.64	17.19	JA-sti
36	ERF	ERF109	Q9SZ06	Ath	8.9E-27	83.82	111.60	10.30	14.75	1.71	4.52	JA-sti
37	WRKY	WRKY33	Q8S8P5	Ath	3.6E-51	87.74	5.58	1.30	0.57	0.46	2.10	JA-sti
38	WRKY	WRKY33	Q8S8P5	Ath	3.6E-51	87.74	1.92	0.87	0.50	0.26	0.75	JA-sti
23	bHLH	BHLH13	Q9LNJ5	Ath	1.1E-86	70.62	24.28	5.51	15.05	12.89	14.44	JA-sti
24	ERF	ERF109	Q9SZ06	Ath	6.7E-25	80.88	64.11	7.22	1.88	0.32	0.81	SA-sti
25	MYB_related	RVE1	F4KGY6	Ath	1.4E-18	86	8.80	2.50	2.97	3.56	4.58	SA-sti
28	MYB	ODO1	Q50EX6	Phy	4.7E-66	86.05	22.68	7.22	6.52	14.36	21.03	SA-sti
39	Dof	DOF1.7	O82155	Ath	8E-30	88.71	38.58	13.28	13.06	8.86	7.78	SA-sti
3	MYB	MYB44	Q9FDW1	Ath	5.4E-33	77.78	30.44	5.77	9.63	2.70	1.65	SA-sti
8	MYB	MYB44	Q9FDW1	Ath	1.2E-28	74.03	10.08	2.37	0.36	0.00	0.00	SA-sti
9	MYB	MYB44	Q9FDW1	Ath	2.4E-16	78	59.04	10.26	21.91	5.89	4.06	SA-sti
40	WRKY	WRKY53	Q9SUP6	Ath	1.3E-40	73.27	49.19	11.98	6.12	8.58	23.58	SA-sti
12	MYB	MYB44	Q9FDW1	Ath	2.4E-48	81.9	75.94	16.12	7.40	1.37	2.61	SA-sti
29	MYB	ODO1	Q50EX6	Phy	2.2E-72	95.35	10.64	1.88	0.75	0.23	2.01	SA-sti
13	WRKY	WRKY40	Q9SAH7	Ath	4E-29	92.19	92.23	19.30	36.11	27.31	49.44	SA-sti
14	WRKY	WRKY70	Q9LY00	Ath	1.6E-17	62.71	51.75	18.71	35.62	40.34	33.77	SA-sti
41	WRKY	WRKY53	Q9SUP6	Ath	2.5E-22	69.7	54.21	10.95	8.69	7.42	16.86	SA-sti
15	MYB	MYB44	Q9FDW1	Ath	1.1E-50	85.71	146.82	26.67	17.47	5.88	7.90	SA-sti
16	NAC	NAC102	Q8H115	Ath	5.9E-26	90	63.31	18.15	8.28	23.77	51.21	SA-sti
17	NAC	NAC102	Q8H115	Ath	1.6E-51	90.1	30.93	9.31	3.19	8.77	28.20	SA-sti
18	NAC	NAC002	Q39013	Ath	1.7E-21	88.46	200.17	30.53	26.42	32.49	83.49	SA-sti
31	NAC	NAC102	Q8H115	Ath	1.3E-21	90.38	318.41	81.88	49.04	78.02	101.43	SA-sti
19	NAC	NAC002	Q39013	Ath	2.3E-44	90.7	601.19	127.24	89.40	102.27	186.80	SA-sti
20	NAC	NAC102	Q8H115	Ath	4.8E-51	90.91	104.53	27.28	22.25	28.75	76.53	SA-sti
6	WRKY	–	–	–	–	–	331.73	40.78	75.34	6.91	98.37	SA-sti
35	WRKY	WRKY54	Q93WU8	Ath	2.1E-13	77.27	144.39	27.50	27.56	10.64	17.19	SA-sti
36	ERF	ERF109	Q9SZ06	Ath	8.9E-27	83.82	111.60	10.30	14.75	1.71	4.52	SA-sti
37	WRKY	WRKY33	Q8S8P5	Ath	3.6E-51	87.74	5.58	1.30	0.57	0.46	2.10	SA-sti
38	WRKY	WRKY33	Q8S8P5	Ath	3.6E-51	87.74	1.92	0.87	0.50	0.26	0.75	SA-sti
10	MYB	MYB44	Q9FDW1	Ath	1.1E-52	89.52	97.00	36.58	67.57	44.99	66.88	SA-sti
40	WRKY	WRKY53	Q9SUP6	Ath	1.3E-40	73.27	49.19	11.98	6.12	8.58	23.58	ROS
42	NAC	NAC053	Q949N0	Ath	5.9E-09	75.76	37.83	16.34	19.75	19.77	21.22	ROS
43	HSF	HSFB2B	Q9T0D3	Ath	3.2E-50	90	103.40	30.71	27.61	38.43	58.95	ROS
44	ERF	DREB2A	O82132	Ath	1.9E-37	85.71	36.79	13.78	14.76	11.20	12.52	ROS
45	ERF	ERF105	Q8VY90	Ath	1.3E-27	83.33	3.18	0.14	0.00	0.00	3.52	ROS
46	HSF	HSF30	P41152	Spe	5.8E-75	80.62	120.47	52.36	22.89	65.28	53.76	ROS
47	HSF	HSF21	O49403	Ath	5.4E-50	83.18	13.34	7.87	6.71	5.42	9.58	ROS
41	WRKY	WRKY53	Q9SUP6	Ath	2.5E-22	69.7	54.21	10.95	8.69	7.42	16.86	ROS
48	NAC	NAC016	A4FVP6	Ath	1.3E-21	60.27	22.51	9.29	14.82	16.99	15.15	ROS
49	NAC	NAC016	A4FVP6	Ath	9.2E-84	77.9	30.53	12.92	21.63	20.84	25.27	ROS
35	WRKY	WRKY54	Q93WU8	Ath	2.1E-13	77.27	144.39	27.50	27.56	10.64	17.19	ROS
50	HSF	HSFA2	O80982	Ath	2.4E-31	81.08	115.81	4.64	2.15	4.65	4.53	ROS
c6												
51	NAC	NAC019	Q9C932	Ath	3.6E-85	85.12	4.36	1.69	30.85	114.22	142.70	JA-m
52	NAC	NAC019	Q9C932	Ath	3.6E-85	85.12	1.55	0.00	0.03	0.23	22.32	JA-m
53	MYB	MYB108	Q9LDE1	Ath	5.8E-75	85.43	0.32	0.10	0.34	36.28	187.49	JA-m
54	ERF	ERF1B	Q8LDC8	Ath	3.4E-41	78.22	3.46	0.08	0.69	16.55	44.22	JA-m
55	ERF	ERF1B	Q8LDC8	Ath	1.3E-38	80	1.44	0.00	0.11	2.25	10.34	JA-m
56	ERF	ERF4	Q40477	Nta	3.5E-30	96.88	70.54	33.95	65.84	68.46	120.33	JA-m
57	NAC	NAC019	Q9C932	Ath	1.2E-85	85.71	37.31	5.38	17.62	150.39	288.55	JA-m
58	NAC	NAC029	O49255	Ath	5.9E-43	85.11	2.90	2.62	17.95	62.24	119.67	JA-m
59	MYB	MYB305	P81391	Ama	2E-45	76.47	0.93	0.35	1.68	3.47	35.34	JA-m
60	ERF	PTI5	O04681	Sly	1E-27	90.91	2.90	4.55	5.68	3.55	19.16	JA-m
61	HB-other	OCP3	Q8H0V5	Ath	2.6E-26	76	1.69	1.53	1.67	4.32	10.16	JA-m
62	HB-other	OCP3	Q8H0V5	Ath	2.6E-26	76	2.16	2.69	2.90	7.83	13.35	JA-m
63	ERF	ERF3	Q9SXS8	Nta	6.2E-32	86.08	43.94	44.21	57.46	94.23	125.35	JA-m
64	MYB	MYB108	Q9LDE1	Ath	4.2E-52	70.54	0.00	0.08	0.49	7.82	35.21	JA-m
65	NAC	NAC019	Q9C932	Ath	1.7E-06	95.65	1.70	0.12	2.06	9.84	16.33	JA-m
66	WRKY	WRKY51	Q93WU9	Ath	1.8E-29	67.47	1.08	0.55	2.17	4.61	12.15	JA-m
67	MIKC_MADS	MADS25	Q84NC5	Osa	3.5E-11	64.15	2.17	1.92	2.31	9.26	17.84	JA-m
68	C2H2	IDD1	Q9LVQ7	Ath	1.7E-93	94.48	14.36	13.96	19.01	24.23	31.01	JA-m
69	C3H	AtC3H20	O82199	Ath	5.8E-58	78.07	9.20	2.60	11.27	11.99	35.91	JA-m
70	NAC	NAC019	Q9C932	Ath	1.7E-06	95.65	0.50	0.00	3.42	5.85	10.84	JA-m
71	MIKC_MADS	J	Q9FUY6	Sly	1.7E-76	84.66	15.08	5.34	1.89	65.59	152.35	JA-m
72	NAC	NAC48	Q7F2L3	Osa	1.3E-36	85	8.37	3.97	0.27	6.78	28.98	r-ABA-m
73	ERF	ERF110	Q70II3	Ath	1.2E-32	92.65	0.58	0.81	3.43	5.82	14.80	r-ABA-m
74	HD-ZIP	ATHB-5	P46667	Ath	9.9E-41	78	92.18	30.07	38.30	136.87	204.33	r-ABA-m
61	HB-other	OCP3	Q8H0V5	Ath	2.6E-26	76	1.69	1.53	1.67	4.32	10.16	r-ABA-m
62	HB-other	OCP3	Q8H0V5	Ath	2.6E-26	76	2.16	2.69	2.90	7.83	13.35	r-ABA-m
65	NAC	NAC019	Q9C932	Ath	1.7E-06	95.65	1.70	0.12	2.06	9.84	16.33	r-ABA-m
70	NAC	NAC019	Q9C932	Ath	1.7E-06	95.65	0.50	0.00	3.42	5.85	10.84	r-ABA-m
75	ERF	ERF110	Q70II3	Ath	1E-27	80.88	0.46	0.13	0.28	2.07	15.42	r-ABA-m
76	NAC	NAC002	Q39013	Ath	1.2E-85	92.31	17.10	2.01	32.10	296.37	442.02	r-ABA-m
77	ERF	ERF110	Q70II3	Ath	1.9E-35	88.31	0.00	0.14	0.74	1.74	2.45	r-ABA-m
51	NAC	NAC019	Q9C932	Ath	3.6E-85	85.12	4.36	1.69	30.85	114.22	142.70	PCD
52	NAC	NAC019	Q9C932	Ath	3.6E-85	85.12	1.55	0.00	0.03	0.23	22.32	PCD
71	MIKC_MADS	J	Q9FUY6	Sly	1.7E-76	84.66	15.08	5.34	1.89	65.59	152.35	PCD
78	NAC	NAC100	Q9FLJ2	Ath	4.6E-45	82.47	15.08	5.34	1.89	65.59	152.35	PCD
79	bZIP	TGA3	Q39234	Ath	2.4E-48	66.67	3.14	1.76	3.33	14.19	23.11	PCD
80	LSD	LSD1	P94077	Ath	1.1E-35	72.04	1.33	0.92	0.55	6.04	22.95	PCD
67	MIKC_MADS	MADS25	Q84NC5	Osa	3.5E-11	64.15	2.17	1.92	2.31	9.26	17.84	PCD
81	NAC	NAC100	Q9FLJ2	Ath	1.4E-71	79.87	2.17	1.92	2.31	9.26	17.84	PCD
82	LSD	LSD1	P94077	Ath	7.5E-39	68.93	6.17	8.43	13.47	41.89	55.71	PCD
83	LSD	LSD1	P94077	Ath	1.1E-35	72.04	1.18	1.67	2.11	2.02	3.68	PCD
84	NAC	NAC100	Q9FLJ2	Ath	4.2E-88	81.46	4.23	5.69	9.40	8.64	19.69	PCD
85	bZIP	CPRF2	Q99090	Pcr	1.3E-38	86.96	5.98	4.71	9.10	14.13	20.20	PCD
86	bZIP	TGA4	Q39162	Ath	1.2E-47	67.91	25.15	22.56	32.93	38.52	49.17	PCD
87	bZIP	TGA21	O24160	Nta	2E-138	81.25	3.97	5.36	6.18	12.28	14.62	PCD
88	LSD	LSD1	P94077	Ath	1.1E-35	72.04	19.46	17.22	26.41	36.47	45.97	PCD
68	C2H2	IDD1	Q9LVQ7	Ath	1.7E-93	94.48	14.36	13.96	19.01	24.23	31.01	PCD
89	TALE	BLH1	Q9SJ56	Ath	3.2E-52	94.29	14.36	13.96	19.01	24.23	31.01	PCD
90	NAC	NAC100	Q9FLJ2	Ath	2.6E-79	79.88	17.04	10.57	32.60	62.18	82.40	PCD

“-” means no corresponding annotation in Swiss-Prot database. *Abbreviation*: *Ath Arabidopsis thaliana*, *Nsy Nicotiana sylvestris*, *Phy Petunia hybrid*, *Osa Oryza sativa subsp. japonica*, *Spe Solanum peruvianum*, *Nta Nicotiana tabacum*, *Ama Antirrhinum majus*, *Sly Solanum lycopersicum*, *Pcr Petroselinum crispum*, *ABA-m* ABA mediated signaling pathway, *SA-sti* response to SA stimulus, *JA-sti* response to JA stimulus, *JA-m* JA mediated signaling pathway, *r-ABA-m* regulation of ABA mediated signaling pathway

## Discussion

### Comprehensive analysis of TF expression profiles throughout *L. japonica* flower development

TFs are proteins that bind specific DNA sequences and regulate transcription. Recent literature demonstrated that TFs play significant roles in the growth and development of plants [43]. In *A.thaliana* genomes, nearly 6% of genes encode TFs [44]. Nevertheless, our knowledge on TFs in non-model plants are limited. In this study, TFs in five different developmental stages of *L. japonica* flower were investigated in order to better understand the roles of TFs *L. japonica* flower. To ensure the accuracy of identification and classification of TFs, the unigenes of *L. japonica* flower were aligned with the amino acid sequences of two other species including *A.thaliana* and *C.canephora*. Our results showed that 1316 TFs were identified and classified into 52 families (Fig. 2a, Fig. 3a). According to PlantTFDB, in *A.thaliana*, there were 2296 TFs (1717 loci) that belonging to 58 families. In *C.canephora*, which has a closer relationship to *L. japonica*, 1256 TFs (1256 loci) were identified and classified into 57 families [31].

Each stage of the *L. japonica* flower is characterized by different properties, which may be related to the regulatory activities of TFs. In our study, the accumulations of CGA and luteoloside, the most important active components in *L. japonica*, were variable at different stages (Fig. 1). Expression profiles of TFs were analyzed at the five different stages: S1, S2, S3, S4 and S5. Among the 1316 total TFs, 1279, 1275, 1268, 1241 and 1254 TFs were expressed in S1, S2, S3, S4 and S5, respectively (Fig. 2b). Additionally, 12 stage-specific TFs were found at S5. These 12 TFs included 3 ERF (ERF3), 3 SBP (SPL1), 2 YABBY (YAB2), 2 MIKC_MADS (AGL8), 1 GRAS (SCL15), 1 HD-ZIP(HAT7) (Table 3). SPL1 plays an important role in inflorescence [45], YAB2 is expressed in floral organ primordium and SCL15 represses embryonic traits in seeding acting [46, 47]. All of these TFs are related to the developmental processes in flowers, indicating that they may play important regulatory roles in S5.

Among the 52 families in the total 1316 TFs, the five largest families were bHLH (108), ERF (95), MYB (89), bZIP (75), and MYB-related (66) (Fig. 3a). All of these families are large TF families in plants and are involved in the regulation of numerous biological processes [48, 49]. As for the 917 DETFs, the five largest families were bHLH (86), ERF (71), MYB (72), bZIP (49), and NAC (46) (Fig. 3a). Compared with the five largest families of the total TFs, bHLH, ERF, MYB, bZIP were the constant, while NAC replaced MYB-related as the fifth largest family of the DETFs. The NAC family is the second-largest TF family in plant, and the TFs in NAC regulate diverse processes, such as floral development and auxin signaling [50]. This implies that the TFs in NAC family are active and may have significant roles in regulatory activities during the development of *L. japonica* flower.

### The transcriptional regulation of active compounds biosynthesis by TFs in *L. japonica* flower

CGA and luteoloside are the standard chemicals for evaluating the chemical quality of *L. japonica* [10]. In this study, the content of CGA was higher at S2 and S3, and the content of luteoloside was highest at S3 (Fig. 1). CGA is a major member of soluble plant phenolics [51], and luteolin is an important plant flavonoid [52]. Both biosynthesis of CGA and luteolin derive from the phenylpropanoid pathway. Phenylpropanoids comprise an important class of plant secondary metabolites. A number of TFs have been reported to regulate branches of phenylpropanoid metabolism [53]. MYB–bHLH–WDR protein complexes (MBW) that consist of TFs of MYB and bHLH families and WD40 proteins regulate phenylpropanoids biosynthesis [54]. This process is highly conserved in higher plants [20]. In this study, most of the TFs involved in flavonoid pathway belonged to bHLH or MYB families, indicating that the important roles of these complexes in *L. japonica* flower (Additional file 3: Table S2, Additional file 1: Figure S5).

TFs MYB114 and WRKY44 are members of MBW. Previous study demonstrated that MYB114 increases accumulation of anthocyanin by activating the expression of anthocyanin biosynthesis genes [20]. WRKY44, also known as TTG2, controls proanthocyanidin biosynthesis [55]. In this study, MYB114 belonged to c1 cluster, the expression of it peaked at S3 and exhibited a high correlation with the content of luteoloside, while WRKY44 belonged to c2 cluster, the expression of it decreased from S1 to S5 (Figs. 1 and 4). These results indicate that MYB114 may play an important role in luteoloside biosynthesis and the regulatory mechanisms of MBW in *L. japonica* flower are complex.

Moreover, MYB12, also known as PFG1, is a member of MBW, too [56]. It has been reported that MYB12 is involved in phenylpropanoid pathway in *Arabidopsis*, tobacco, and tomato plants, acting as a very effective and positive regulator in the biosynthesis of caffeoyl quinic acids and flavonols [19]. CHS and FLS are key enzymes in the phenylpropanoid pathway. MYB12 specifically activates the flavonol pathway by inducing the expression of CHS and FLS [57]. *MYB12* and *CHS* shows same transcription profiles in *Asiatic hybrid lily* during its flower bud development [58]. In tomato plants, AtMYB12 leads to the induction of the biosynthetic genes required for the production of flavonols and CGA [19]. In this study, the expression of *MYB12*, *CHS* and *FLS* was found to be highly consistent with each other (Fig. 9a). This result indicates that, in *L. japonica*, MYB12 may play a significant role in controlling the expression of *CHS* and *FLS* in different developmental processes. On the other hand, flavonoid biosynthesis is also controlled by negative regulation as the repression of JA-mediated defense by MYB44 contributes to the low accumulation [59]. The expression of *MYB12*, *CHS*, and *FLS* was maintained higher levels in S2, while the expression of *MYB44* decreased in S2 (Fig. 9a). This result indicates that MYB44 may have a negative role in regulating the gene expression of *CHS* and *FLS* in *L. japonica*.

Besides, antioxidants have emerged as prophylactic and therapeutic agents for various diseases. Previous study demonstrated that extracts from the *L. japonica* flower exhibit antioxidative activity [6]. However, little is known about the transcriptional regulation concerning the antioxidant properties of *L. japonica*. LOL1, LOL2 and LSD1 belong to LSD TF family. LSD1 can positively regulate the accumulation of SOD, which is an enzyme capable of scavenging O2˙- and leading to H_2_O_2_ formation [60, 61]. On the contrary, LOL1 and LOL2 play negative roles in SOD accumulation [61]. In this study, the expression of *LSD1* and *SOD*, the content of H_2_O_2_ and the total antioxidant capacity were positively correlated, while negatively correlating with the expression of *LOL1* and *LOL2* (Fig. 9c). Our results were in accordance with those from previous researches, indicating the regulatory roles of LSD1 LOL1, and LOL2 on antioxidation in *L. japonica* flower.

### The transcription regulation of PCD by TFs in NAC and WRKY families in *L. japonica* flower

The mechanisms of plant developmental PCD have been extensively studied [62]. Flowers have a species-specific, limited life span with an irreversible program of senescence. To some degree, the terms ‘senescence’ and ‘PCD’ are equivalent for flowers [63]. NAC and WRKY TFs have been closely associated with senescence in several tissues such as *Arabidopsis* leaves, petals and siliques, and they interact with many regulators including plant hormones and ROS [17, 64]. Plant hormones like JA, SA, and ABA act as positive regulators of plant senescence [17, 65]. ROS have been proposed as key inducers of developmental PCD or senescence [40]. In *L. japonica* flower, many TFs known to involve in PCD or senescence were detected (Table 4). Moreover, our results support the complex relationships between TFs and many regulators like JA, SA, ABA and ROS that are involved in controlling PCD *L. japonica* flower.

In *L. japonica*, many TFs in cluster 6 were associated with PCD (Fig. 6). These TFs belonged to different families, including bZIP (4), NAC (2), MIKC_MADS (2), LSD (1), C2H2 (1), and TALE (1) (Table 4). These families have been reported to play regulatory roles in plant senescence [66–70]. In addition, the expression of these TFs was highest at S5, the last stage before senescence of *L. japonica* flower, indicating that these TFs play important roles in the process of senescence. As shown in Table 4, TFs in NAC family that associated with PCD included NAC19 and NAC29. The expression of NAC19 is strongly induced by ABA, slightly induced by JA, and is known to mediate PCD through ROS accumulation [24]. NAC29 can be induced by ABA and may have a positive role in precocious senescence [71]. In addition to NAC19 and NAC29, we also identified NAC53 in cluster 6. NAC53 promotes ROS production in leaves [72], which may further trigger PCD or senescence of plant. In *L. japonica*, high correlations among the expression of *NAC19*, *NAC29*, *NAC53*, the contents of ABA, JA, and H_2_O_2_ were observed (Fig. 9b). These results support that NAC19, NAC29, NAC53 may be important in the regulatory network involving PCD and hormones in the senescence process of *L. japonica*, a potential topic worthy of further analysis.

Furthermore, several WRKY TFs involved in regulation of leaf senescence and response to hormones were also identified in *L. japonica* flower (Table 4). WRKY70 and WRKY54 are regulated by SA and JA, and co-operate as negative regulators of senescence [73]. In this study, the expression of *WRKY70* and *WRKY54* were low at S3, S4 and S5, while the contents of SA and JA, the positive regulators of plant senescence, were high at S3, S4 and S5 (Figs. 4 and 7). This implied the negative regulation by WRKY 70 and WRKY 54 on senescence in *L. japonica* flower. Additionally, WRKY53 is modulated by the JA and SA equilibrium in a complex TF signaling network that regulates senescence [25, 74]. It has been reported that WRKY53, WRKY54, and WRKY70 might be involved in a regulatory network that integrates internal and environmental signals to modulate the onset and the development of leaf senescence [73]. In this study, our results indicate that this regulatory network may also have significant roles in *L. japonica* flower.

Taken together, the expression of TFs and the presence of different flower development regulators, including ABA, JA, SA and ROS, as well as their interactions, demonstrate complexity of the regulatory network involved in flower development. Further investigations on the various associations between these elements are essential to better understand the potential mechanisms involved in the medicinal effect of *L. japonica* flower.

## Conclusion

TFs play significant roles during plant growth and development. To better understand the TFs’ regulatory roles in *L. japonica* flower, we aim to comprehensively characterize the expression profiles of TFs at different developmental stages of *L. japonica* flower. General analysis identified 1316 TFs that were classified into 52 families. The largest families included bHLH (108), ERF (95), MYB (89), bZIP (75), and MYB-related (66). S5, the last developmental stage investigated in this study, possessed the most stage-specific TFs. From the DETFs analysis, TFs from bHLH, ERF, MYB, bZIP, and NAC families exhibited obviously altered expression during the growth of flower. Moreover, cluster and GO enrichment analyses were performed on the DETFs, and 6 clusters were grouped. Many GO terms associated with JA, SA, ABA, ROS and PCD were enriched in cluster 4 and cluster 6. In addition, several significant TFs involved in the biosynthesis of active components, the antioxidant activity, and the development of the *L. japonica* flower were detected based on the enrichment results. In phenylpropanoids biosynthesis of *L. japonica* flower, MYB114, WRKY44, MYB12 and MYB44 may have regulatory roles. TFs in LSD family, including LOL1, LOL2, and LSD1, were found to exhibit potentially antagonistic effects on SOD accumulation and antioxidation in *L. japonica* flower. During the flower senescence processes, TFs in NAC and WRKY families, including NAC19, NAC29, NAC53, WRKY70, WRKY54, and WRKY53, may play significant roles in the regulation of hormones. Taken together, our results serve as valuable resource for further studies.

## Additional files

Additional file 1: **Figure S1.** The morphological photos of *L. japonica* flower in five different developmental stages. **Figure S2.** Annotation of assembled *L. japonica* unigenes. In total 28780 unigenes were annotated by different databases, including NR, GO, TrEMBL, Swiss_Prot and Pfam. **Figure S3.** Identification of the transcripts of other plant species homologous to the annotated unigenes of *L. japonica* by NR database. **Figure S4.** Heatmap of DETFs at different developmental stages of *L. japonica* flower. **Figure S5.** Family distribution of TFs in each cluster. (PDF 591 kb)

Additional file 2: **Table S1.** GO enrichment results of each cluster. (XLSX 28 kb)

Additional file 3: **Table S2.** GO annotation involved in flavonoid synthesis in each cluster. (XLSX 15 kb)

## Abbreviations

ABA: Abscisic acid
CGA: Chlorogenic acid
CHS: Chalcone synthase
DETFs: Differentially expressed transcription factors
FLS: Flavonol synthase
GA: Gibberellin
JA: Jasmonic acid
PCD: Programmed cell death
ROS: Reactive oxygen species
SA: Salicylic acid
SOD: Superoxide dismutase
TF: Transcription factor

## References

[1] Yang Y, Song W, Zhu C, Lin S, Zhao F, Wu X, et al. Homosecoiridoids from the flower buds of *Lonicera japonica*. J Nat Prod. 2011;74(10):2151–60. 10.1021/np2004566 21942812

[2] Seung-Hwan K, Shi-Xun M, Sa-Ik H, Seok-Yong L, Choon-Gon J (2015). *Lonicera japonica* THUNB. Extract inhibits lipopolysaccharide-stimulated inflammatory responses by suppressing NF-κB signaling in BV-2 microglial cells. J Med Food.

[3] Ding Y, Cao Z, Cao L, Ding G, Wang Z, Xiao W (2017). Antiviral activity of chlorogenic acid against influenza a (H1N1/H3N2) virus and its inhibition of neuraminidase. Sci Rep.

[4] Ku Sae-Kwang, Seo Bu-Il, Park Ji-Ha, Park Gyu-Yeol, Seo Young-Bae, Kim Jae-Soo, Lee Hyeung-Sik, Roh Seong-Soo (2009). Effect of Lonicerae Flos extracts on reflux esophagitis with antioxidant activity. World Journal of Gastroenterology.

[5] Hye-Jung Y, Hyun-Jung K, Seon SY, Eun-Hee P, Chang-Jin L (2008). Anti-angiogenic, antinociceptive and anti-inflammatory activities of *Lonicera japonica* extract. J Pharm Pharmacol.

[6] Shang X, Pan H, Li M, Miao X, Ding H (2011). *Lonicera japonica* Thunb.: Ethnopharmacology, phytochemistry and pharmacology of an important traditional Chinese medicine. J Ethnopharmacol.

[7] Su D, Li S, Zhang W, Wang J, Wang J, Lv M (2017). Structural elucidation of a polysaccharide from *Lonicera japonica* flowers, and its neuroprotective effect on cerebral ischemia-reperfusion injury in rat. Int J Biol Macromol.

[8] Wang D, Zhao X, Liu Y (2017). Hypoglycemic and hypolipidemic effects of a polysaccharide from flower buds of *Lonicera japonica* in streptozotocin-induced diabetic rats. Int J Biol Macromol.

[9] Zhang L, Long Y, Fu C, Xiang J, Gan J, Wu G (2016). Different gene expression patterns between leaves and flowers in *Lonicera japonica* revealed by transcriptome analysis. Front Plant Sci.

[10] Chinese Pharmacopoeia Commission (2010). The Pharmacopoeia of the People’s Republic of China, 2010 ed.

[11] Kong De-xin, Li Yan-qun, Bai Mei, He Han-jun, Liang Guang-xin, Wu Hong (2017). Correlation between the dynamic accumulation of the main effective components and their associated regulatory enzyme activities at different growth stages in Lonicera japonica Thunb. Industrial Crops and Products.

[12] Qi X, Yu X, Xu D, Fang H, Dong K, Li W (2017). Identification and analysis of CYP450 genes from transcriptome of *Lonicera japonica* and expression analysis of chlorogenic acid biosynthesis related CYP450s. PeerJ..

[13] Rai A, Kamochi H, Suzuki H, Nakamura M, Takahashi H, Hatada T (2017). De novo transcriptome assembly and characterization of nine tissues of *Lonicera japonica* to identify potential candidate genes involved in chlorogenic acid, luteolosides, and secoiridoid biosynthesis pathways. J Nat Med.

[14] Yuan Y, Song L, Li M, Liu G, Chu Y, Ma L (2012). Genetic variation and metabolic pathway intricacy govern the active compound content and quality of the Chinese medicinal plant *Lonicera japonica* thunb. BMC Genomics.

[15] Broun P (2004). Transcription factors as tools for metabolic engineering in plants. Curr Opin Plant Biol.

[16] Vom Endt D, Kijne JW, Memelink J (2002). Transcription factors controlling plant secondary metabolism: what regulates the regulators?. Phytochemistry..

[17] Yang Chang-Qing, Fang Xin, Wu Xiu-Ming, Mao Ying-Bo, Wang Ling-Jian, Chen Xiao-Ya (2012). Transcriptional Regulation of Plant Secondary MetabolismF. Journal of Integrative Plant Biology.

[18] Antonio G, Mingzhe Z, LJ M, LA M (2008). Regulation of the anthocyanin biosynthetic pathway by the TTG1/bHLH/Myb transcriptional complex in *Arabidopsis* seedlings. Plant J.

[19] Jie L, Eugenio B, Lionel H, Adrian P, Ricarda N, Paul B (2008). AtMYB12 regulates caffeoyl quinic acid and flavonol synthesis in tomato: expression in fruit results in very high levels of both types of polyphenol. Plant J.

[20] Xu W, Dubos C, Lepiniec L (2015). Transcriptional control of flavonoid biosynthesis by MYB–bHLH–WDR complexes. Trends Plant Sci.

[21] Marsch-Martínez N, de Folter S (2016). Hormonal control of the development of the gynoecium. Curr Opin Plant Biol.

[22] Gan E-S, Huang J, Ito T (2013). Functional roles of histone modification, chromatin remodeling and micrornas in *Arabidopsis* flower development. Int Rev Cell Mol Biol.

[23] Chaiwanon J, Wang W, Zhu J-Y, Oh E, Wang Z-Y (2016). Information integration and communication in plant growth regulation. Cell..

[24] Wang B, Guo X, Wang C, Ma J, Niu F, Zhang H (2015). Identification and characterization of plant-specific NAC gene family in canola (*Brassica napus* L.) reveal novel members involved in cell death. Plant Mol Biol.

[25] Miao Y, Laun T, Zimmermann P, Zentgraf U (2004). Targets of the WRKY53 transcription factor and its role during leaf senescence in *Arabidopsis*. Plant Mol Biol.

[26] Cui X, Ma Z, Tian Q (2018). The relationship between floral organ development period and effective component content of *Lonicera japonica*. Chin Agric Sci Bull.

[27] Yang Bingxian, Guan Qijie, Tian Jingkui, Komatsu Setsuko (2017). Transcriptomic and proteomic analyses of leaves from Clematis terniflora DC. under high level of ultraviolet-B irradiation followed by dark treatment. Journal of Proteomics.

[28] Li H, Durbin R (2009). Fast and accurate short read alignment with burrows–wheeler transform. Bioinformatics..

[29] Grabherr MG, Haas BJ, Yassour M, Levin JZ, Thompson DA, Amit I (2011). Full-length transcriptome assembly from RNA-Seq data without a reference genome. Nat Biotechnol.

[30] Conesa A, Götz S, García-Gómez JM, Terol J, Talón M, Robles M (2005). Blast2GO: a universal tool for annotation, visualization and analysis in functional genomics research. Bioinformatics..

[31] Jin Jinpu, Tian Feng, Yang De-Chang, Meng Yu-Qi, Kong Lei, Luo Jingchu, Gao Ge (2016). PlantTFDB 4.0: toward a central hub for transcription factors and regulatory interactions in plants. Nucleic Acids Research.

[32] Camacho C, Coulouris G, Avagyan V, Ma N, Papadopoulos J, Bealer K (2009). BLAST+: architecture and applications. BMC Bioinformatics.

[33] Mortazavi A, Williams BA, McCue K, Schaeffer L, Wold B (2008). Mapping and quantifying mammalian transcriptomes by RNA-Seq. Nat Meth.

[34] Howe EA, Sinha R, Schlauch D, Quackenbush J (2011). RNA-Seq analysis in MeV. Bioinformatics..

[35] Du Z, Zhou X, Ling Y, Zhang Z, Su Z (2010). agriGO: a GO analysis toolkit for the agricultural community. Nucleic Acids Res.

[36] Pan X, Welti R, Wang X (2008). Simultaneous quantification of major phytohormones and related compounds in crude plant extracts by liquid chromatography–electrospray tandem mass spectrometry. Phytochemistry..

[37] Bradford MM (1976). A rapid and sensitive method for the quantitation of microgram quantities of protein utilizing the principle of protein-dye binding. Anal Biochem.

[38] Qu Li-Jia, Zhu Yu-Xian (2006). Transcription factor families in Arabidopsis: major progress and outstanding issues for future research. Current Opinion in Plant Biology.

[39] Wu ZY, Raven PH. Flora of China. Beijing: Science Press; 2011; vol.19.

[40] DPM VL, DG L (2012). Redox regulation in plant programmed cell death. Plant Cell Environ.

[41] Gechev TS, Hille J (2005). Hydrogen peroxide as a signal controlling plant programmed cell death. J Cell Biol.

[42] Shi Ming-Zhu, Xie De-Yu (2014). Biosynthesis and Metabolic Engineering of Anthocyanins in Arabidopsis thaliana. Recent Patents on Biotechnology.

[43] Franco-Zorrilla José M., Solano Roberto (2017). Identification of plant transcription factor target sequences. Biochimica et Biophysica Acta (BBA) - Gene Regulatory Mechanisms.

[44] Jin J, Zhang H, Kong L, Gao G, Luo J (2014). PlantTFDB 3.0: a portal for the functional and evolutionary study of plant transcription factors. Nucleic Acids Res.

[45] Chao Lu-Men, Liu Yao-Qian, Chen Dian-Yang, Xue Xue-Yi, Mao Ying-Bo, Chen Xiao-Ya (2017). Arabidopsis Transcription Factors SPL1 and SPL12 Confer Plant Thermotolerance at Reproductive Stage. Molecular Plant.

[46] Gao MJ, Li X, Huang J, Gropp GM, Gjetvaj B, Lindsay DL, et al. SCARECROW-LIKE15 interacts with HISTONE DEACETYLASE19 and is essential for repressing the seed maturation programme. Nat Commun. 2015;6:7243. 10.1038/ncomms8243 PMC4507008 26129778

[47] Siegfried KR, Eshed Y, Baum SF, Otsuga D, Drews GN, Bowman JL (1999). Members of the YABBY gene family specify abaxial cell fate in *Arabidopsis*. Development..

[48] Hong JC, Gonzalez DH (2016). Chapter 3 - general aspects of plant transcription factor families. Plant transcription factors.

[49] Mizoi J, Shinozaki K, Yamaguchi-Shinozaki K (2012). AP2/ERF family transcription factors in plant abiotic stress responses. Biochim Biophys Acta.

[50] Olsen AN, Ernst HA, Leggio LL, Skriver K (2005). NAC transcription factors: structurally distinct, functionally diverse. Trends Plant Sci.

[51] CM N (1999). Chlorogenic acids and other cinnamates – nature, occurrence and dietary burden. J Sci Food Agric.

[52] Yuan Y, Wang Z, Jiang C, Wang X, Huang L (2014). Exploiting genes and functional diversity of chlorogenic acid and luteolin biosyntheses in *Lonicera japonica* and their substitutes. Gene..

[53] Zhang Y, Butelli E, Alseekh S, Tohge T, Rallapalli G, Luo J (2015). Multi-level engineering facilitates the production of phenylpropanoid compounds in tomato. Nat Commun.

[54] Chezem WR, Clay NK (2016). Regulation of plant secondary metabolism and associated specialized cell development by MYBs and bHLHs. Phytochemistry..

[55] Zhou M, Memelink J (2016). Jasmonate-responsive transcription factors regulating plant secondary metabolism. Biotechnol Adv.

[56] Xu W, Grain D, Bobet S, Le Gourrierec J, Thévenin J, Kelemen Z (2014). Complexity and robustness of the flavonoid transcriptional regulatory network revealed by comprehensive analyses of MYB–bHLH–WDR complexes and their targets in *Arabidopsis* seed. New Phytol.

[57] Mehrtens F, Kranz H, Bednarek P, Weisshaar B (2005). The *Arabidopsis* transcription factor MYB12 is a flavonol-specific regulator of phenylpropanoid biosynthesis. Plant Physiol.

[58] Lai YS, Shimoyamada Y, Nakayama M, Yamagishi M. Pigment accumulation and transcription of LhMYB12 and anthocyanin biosynthesis genes during flower development in the Asiatic hybrid lily (*Lilium* spp.). Plant Sci (Amsterdam, Neth). 2012;193-194:136–47. 10.1016/j.plantsci.2012.05.013 22794927

[59] Jung C, Shim JS, Seo JS, Lee HY, Kim CH, Choi YD (2010). Non-specific phytohormonal induction of AtMYB44 and suppression of jasmonate-responsive gene activation in *Arabidopsis thaliana*. Mol Cells.

[60] Alscher RG, Erturk N, Heath LS (2002). Role of superoxide dismutases (SODs) in controlling oxidative stress in plants. J Exp Bot.

[61] Epple P, Mack AA, Morris VRF, Dangl JL (2003). Antagonistic control of oxidative stress-induced cell death in *Arabidopsis* by two related, plant-specific zinc finger proteins. Proc Natl Acad Sci U S A.

[62] Kuriyama H, Fukuda H (2002). Developmental programmed cell death in plants. Curr Opin Plant Biol.

[63] Rogers HJ (2006). Programmed cell death in floral organs: how and why do flowers die?. Ann Bot.

[64] Carol W, J.W. YT, D. SA, Vicky B-W, A. RJ. A molecular and structural characterization of senescing Arabidopsis siliques and comparison of transcriptional profiles with senescing petals and leaves. Plant J 2009;57(4):690–705. 10.1111/j.1365-313X.2008.03722.x 18980641

[65] Rogers HJ, Gunawardena AN, McCabe PF (2015). Senescence-associated programmed cell death. Plant programmed cell death.

[66] Adamczyk BJ, Fernandez DE (2009). MIKC* MADS domain heterodimers are required for pollen maturation and tube growth in *Arabidopsis*. Plant Physiol.

[67] E ZG, Zhang YP, Zhou JH, Wang L (2014). Mini review roles of the bZIP gene family in rice. Genet Mol Res.

[68] Feurtado JA, Huang D, Wicki-Stordeur L, Hemstock LE, Potentier MS, Tsang EWT (2011). The *Arabidopsis* C2H2 zinc finger INDETERMINATE DOMAIN1/ENHYDROUS promotes the transition to germination by regulating light and hormonal signaling during seed maturation. Plant Cell.

[69] Podzimska-Sroka D, O'Shea C, Gregersen PL, Skriver K (2015). NAC transcription factors in senescence: from molecular structure to function in crops. Plants..

[70] Xiaozhen H, Yansha L, Xiaoyan Z, Jianru Z, Shuhua Y (2010). The *Arabidopsis* LSD1 gene plays an important role in the regulation of low temperature-dependent cell death. New Phytol.

[71] Yongfeng G, Susheng G (2006). AtNAP, a NAC family transcription factor, has an important role in leaf senescence. Plant J.

[72] Sangmin L, Joon SP, Hyo-Jun L, Chung-Mo P (2012). A NAC transcription factor NTL4 promotes reactive oxygen species production during drought-induced leaf senescence in *Arabidopsis*. Plant J.

[73] Besseau S, Li J, Palva ET (2012). WRKY54 and WRKY70 co-operate as negative regulators of leaf senescence in *Arabidopsis thaliana*. J Exp Bot.

[74] Miao Y, Zentgraf U (2007). The antagonist function of *Arabidopsis* WRKY53 and ESR/ESP in leaf senescence is modulated by the jasmonic and salicylic acid equilibrium. Plant Cell.

