# Supplementary material for: Transcriptional regulation of Lonicera japonica Thunb. during flower development as revealed by comprehensive analysis of transcription factors
Source: BMC Plant Biol. 2019 May 14;19:198. doi: 10.1186/s12870-019-1803-1 (PMC6518806; doi:10.1186/s12870-019-1803-1)
Supplement: Supplementary file 1 — Figure S1. The morphological photos of L. japonica flower in five different developmental stages. Figure S2. Annotation of assembled L. japonica unigenes. In total 28780 unigenes were annotated by different databases, including NR, GO, TrEMBL, Swiss_Prot and Pfam. Figure S3. Identification of the transcripts of other plant species homologous to the annotated unigenes of L. japonica by NR database. Figure S4. Heatmap of DETFs at different developmental stages of L. japonica flower. Figure S5. Family distribution of TFs in each cluster. (PDF 591 kb) [file 12870_2019_1803_MOESM1_ESM.pdf]

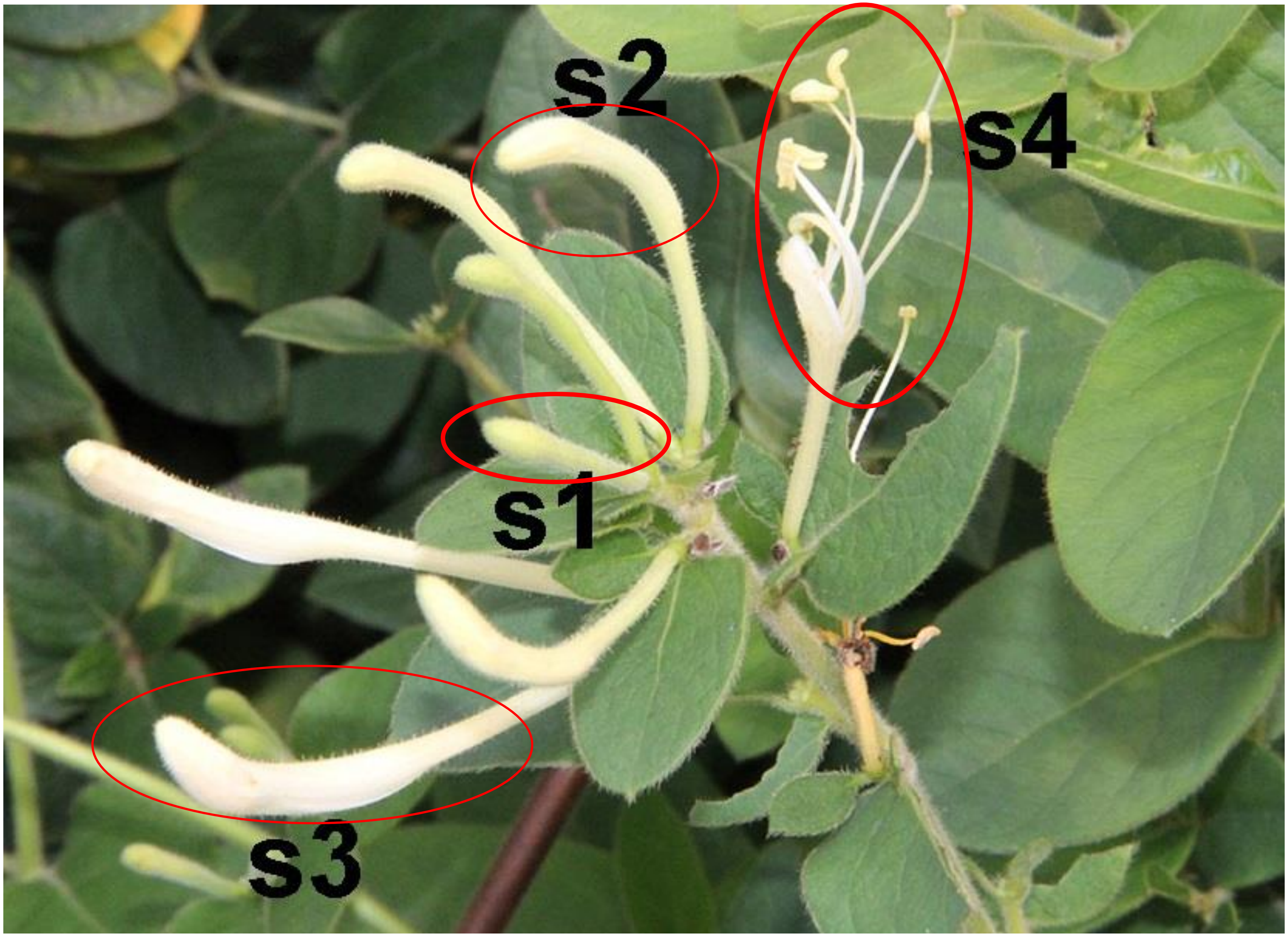
s2
s4
s1
s3

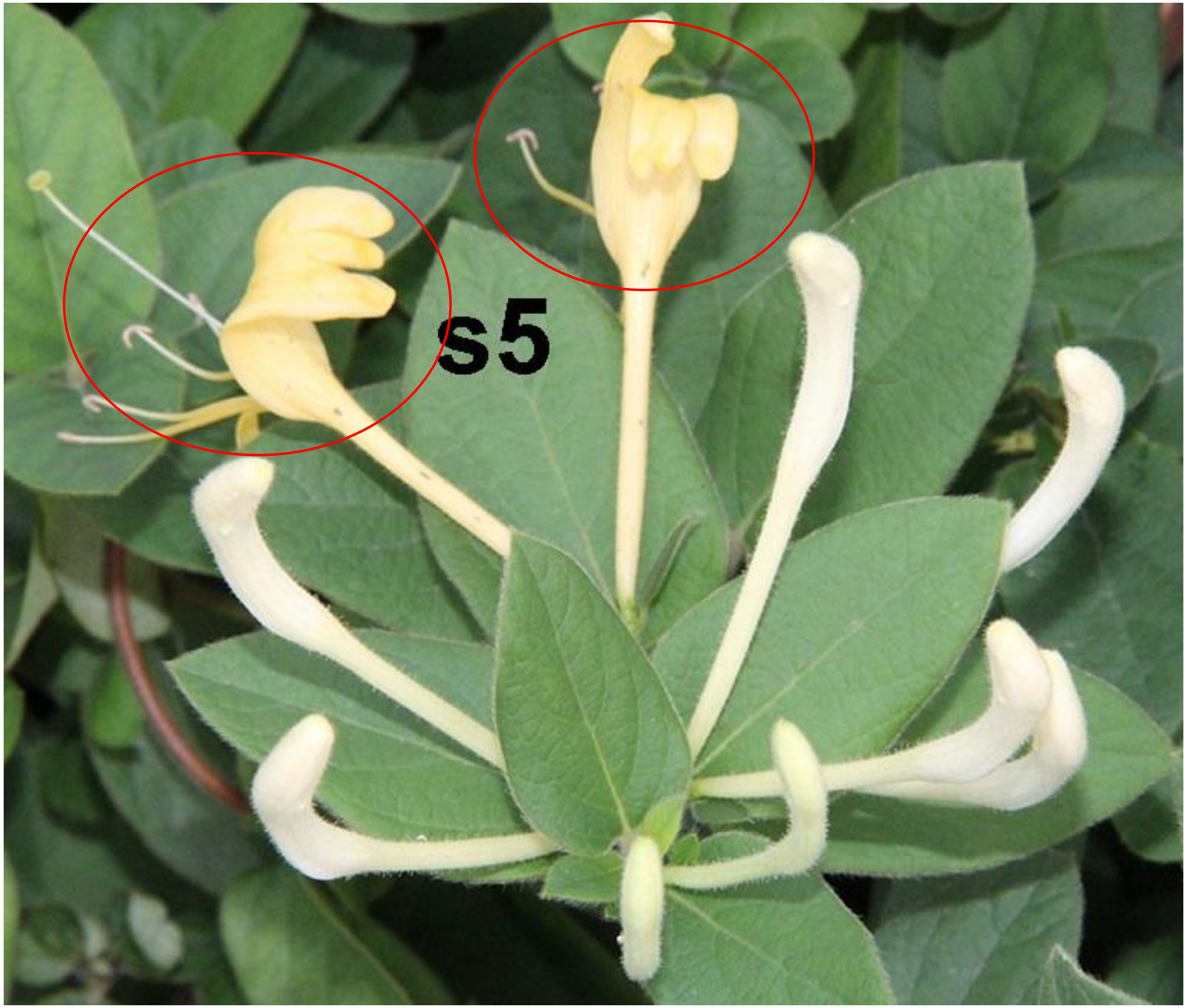

Figure S1 | The morphological photos of *L. japonica* flower in five different developmental stages. S1: The juvenile bud stage, S2: The third green stage, S3: The complete white stage, S4: The silver flowering stage, S5: The gold flowering stage

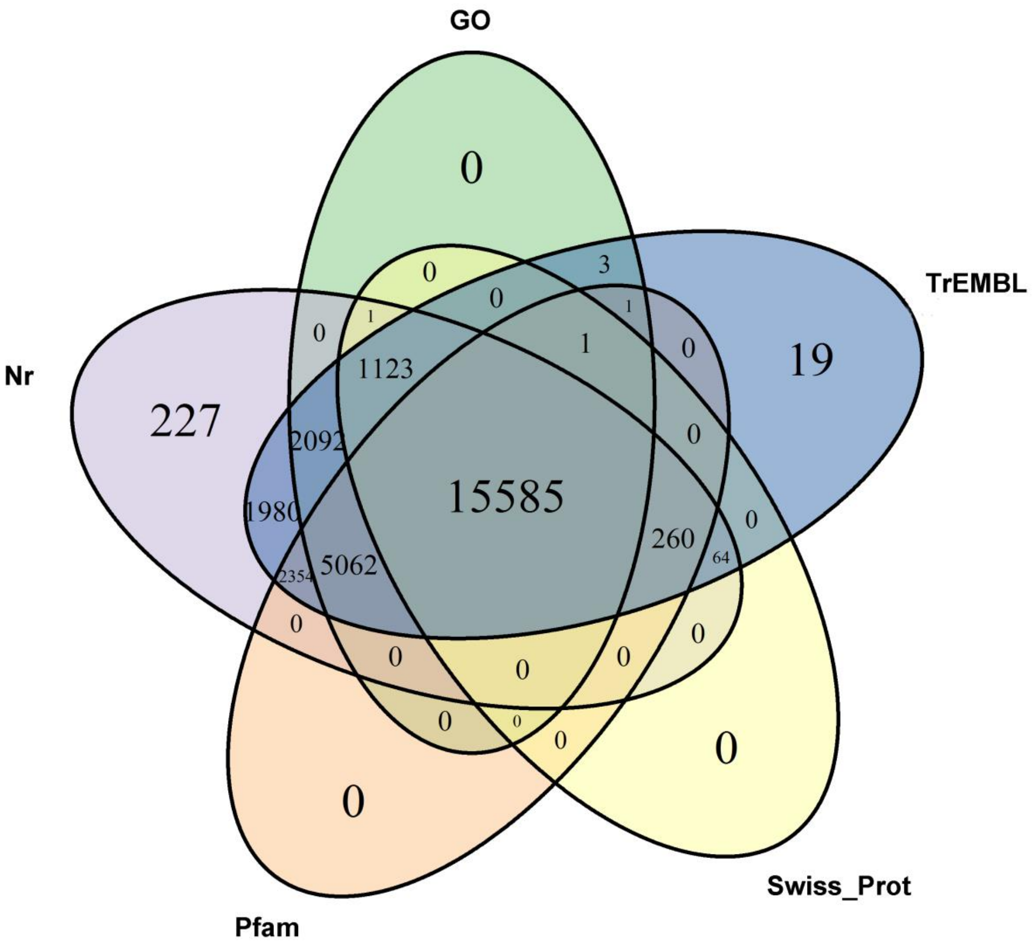

Figure S2 | Annotation of assembled *L. japonica* unigenes. In total, 28780 unigenes were annotated by different databases, including NR, GO, TrEMBL, Swiss_Prot, Pfam. The annotation was conducted using Blast with an E-value cut-off of 1e-5.

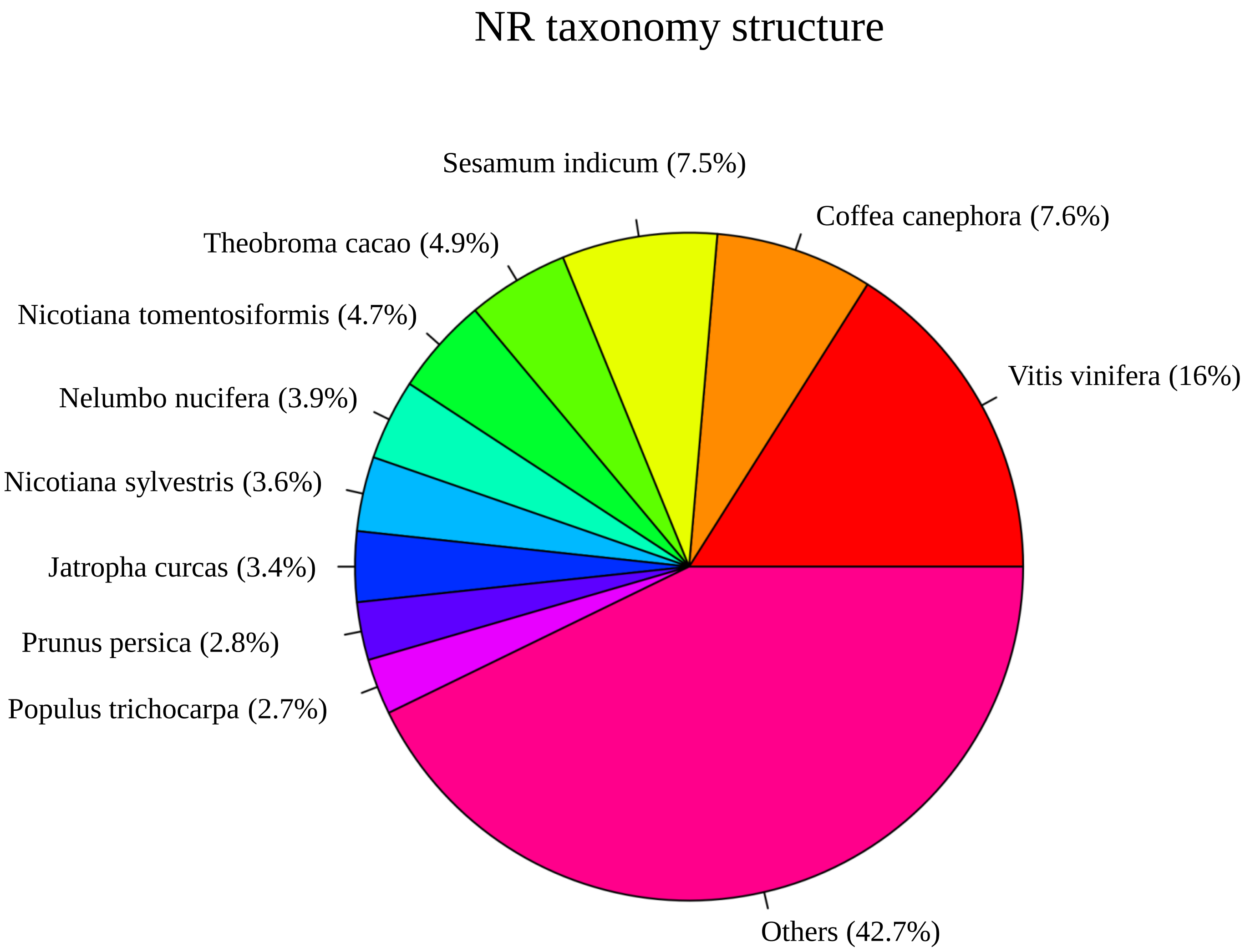

Figure S3 | Identification of the transcripts of other plant species homologous to the annotated unigenes of *L. japonica* in NR database.

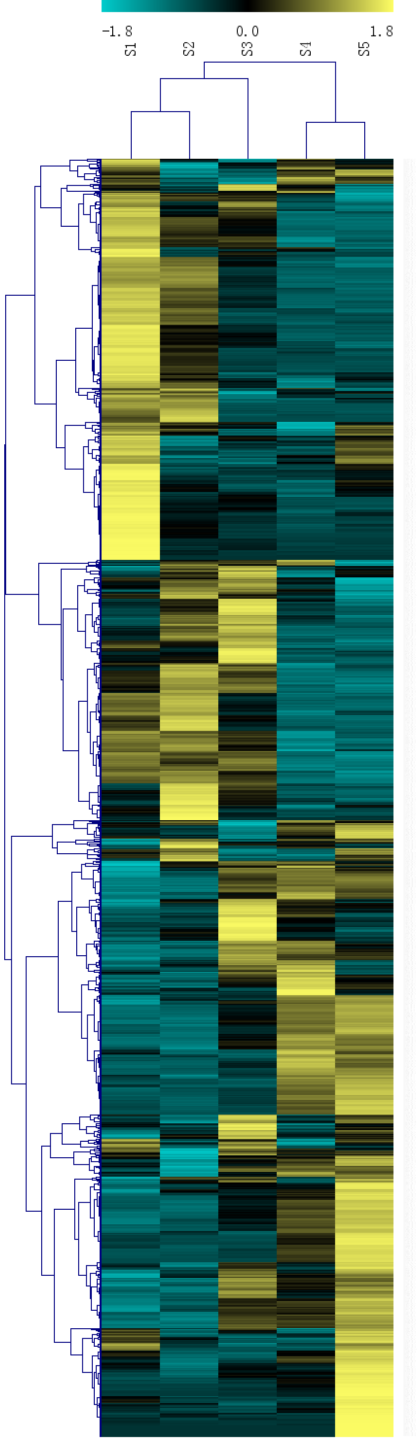

Figure S4 | Heatmap of DETFs in different stages of *L. japonica* flowers.

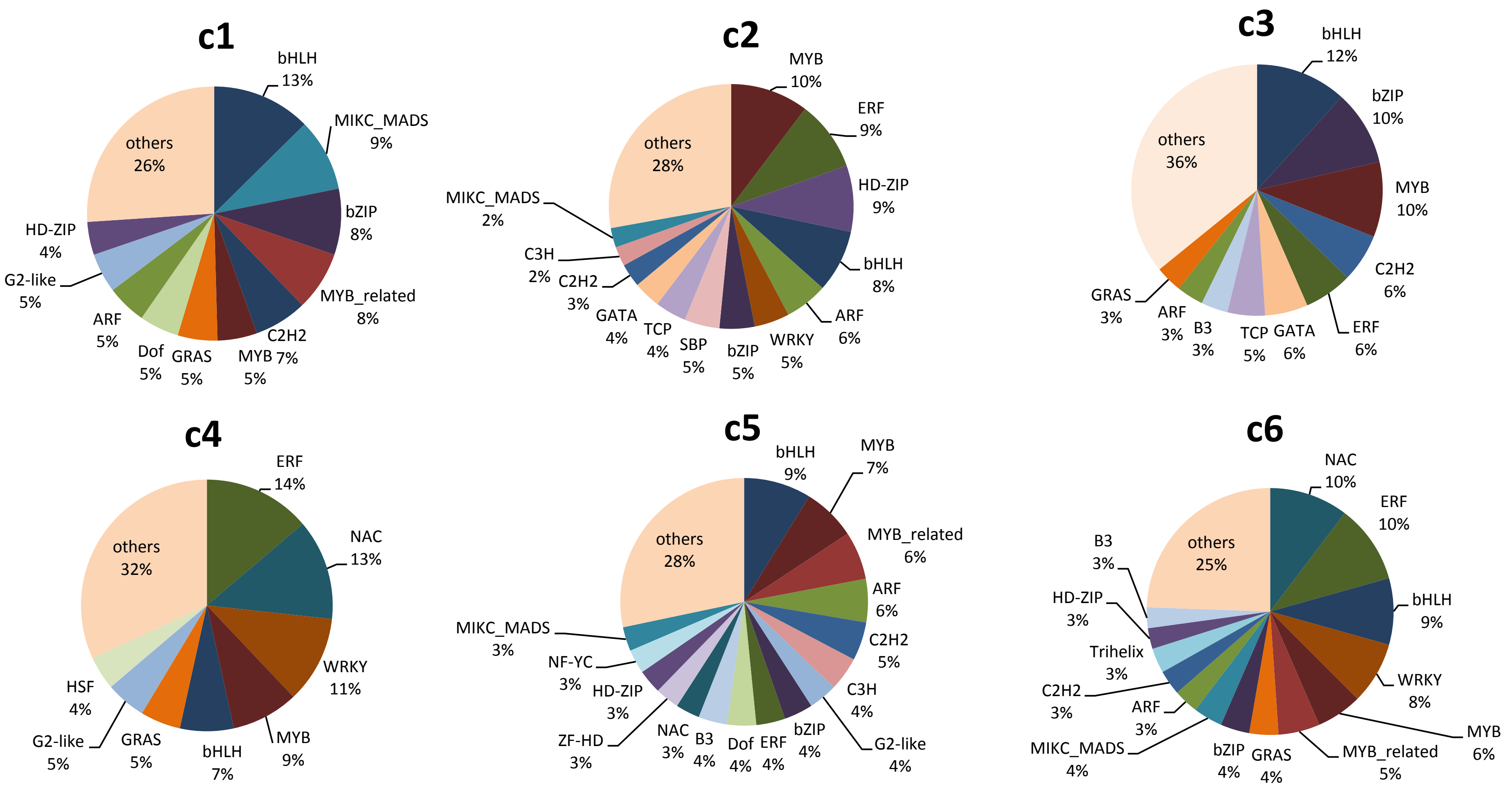

Figure S5 | Family distribution of TFs in each cluster. The TFs family that the members in it less than five was considered as others.
